# GSK-3β suppression upregulates Gli1 to alleviate osteogenesis inhibition in titanium nanoparticle-induced osteolysis

**DOI:** 10.1186/s12951-022-01351-7

**Published:** 2022-03-19

**Authors:** Qing Wang, Wei Zhang, Xiaole Peng, Yunxia Tao, Ye Gu, Wenming Li, Xiaolong Liang, Liangliang Wang, Zerui Wu, Tianhao Wang, Haifeng Zhang, Xin Liu, Yaozeng Xu, Yu Liu, Jun Zhou, Dechun Geng

**Affiliations:** 1 Department of Orthopaedics, The First Affiliated Hospital of Soochow University, Suzhou, 215006 China; 2 Department of Orthopaedics, Wuxi Ninth People’s Hospital Affiliated to Soochow University, Wuxi, 214062 China; 3 Department of Orthopaedics, Changshu Hospital Affiliated to Soochow University, First People’s Hospital of Changshu City, Changshu, China; 4 Department of Orthopaedics, The Affiliated Changzhou No. 2 People’s Hospital of Nanjing Medical University, Changzhou, People’s Republic of China; 5 Department of Orthopaedics, The Affiliated Hospital of Xuzhou Medical University, Xuzhou, China

**Keywords:** Hedgehog signaling pathway, Osteogenesis, Bone formation, Titanium nanoparticles, Periprosthetic osteolysis

## Abstract

Wear particle-induced periprosthetic osteolysis (PPO) have become a major reason of joint arthroplasty failure and secondary surgery following joint arthroplasty and thus pose a severe threat to global public health. Therefore, determining how to effectively suppress particle-induced PPO has become an urgent problem. The pathological mechanism involved in the PPO signaling cascade is still unclear. Recently, the interaction between osteogenic inhibition and wear particles at the implant biological interface, which has received increasing attention, has been revealed as an important factor in pathological process. Additionally, Hedgehog (Hh)-Gli1 is a crucial signaling cascade which was regulated by multiple factors in numerous physiological and pathological process. It was revealed to exert a crucial part during embryonic bone development and metabolism. However, whether Hh-Gli1 is involved in wear particle-induced osteogenic inhibition in PPO remains unknown. Our present study explored the mechanism by which the Hh-Gli1 signaling cascade regulates titanium (Ti) nanoparticle-induced osteolysis. We found that Hh-Gli1 signaling was dramatically downregulated upon Ti particle treatment. Mechanistically, glycogen synthesis kinase 3β (GSK-3β) activation was significantly increased in Ti particle-induced osteogenic inhibition via changes in GSK-3β phosphorylation level and was found to participate in the posttranslational modification and degradation of the key transcription factor Gli1, thus decreasing the accumulation of Gli1 and its translocation from the cytoplasm to the nucleus. Collectively, these findings suggest that the Hh-Gli1 signaling cascade utilizes a GSK3β-mediated mechanism and may serve as a rational new therapeutic target against nanoparticle-induced PPO.

**Graphical Abstract:**

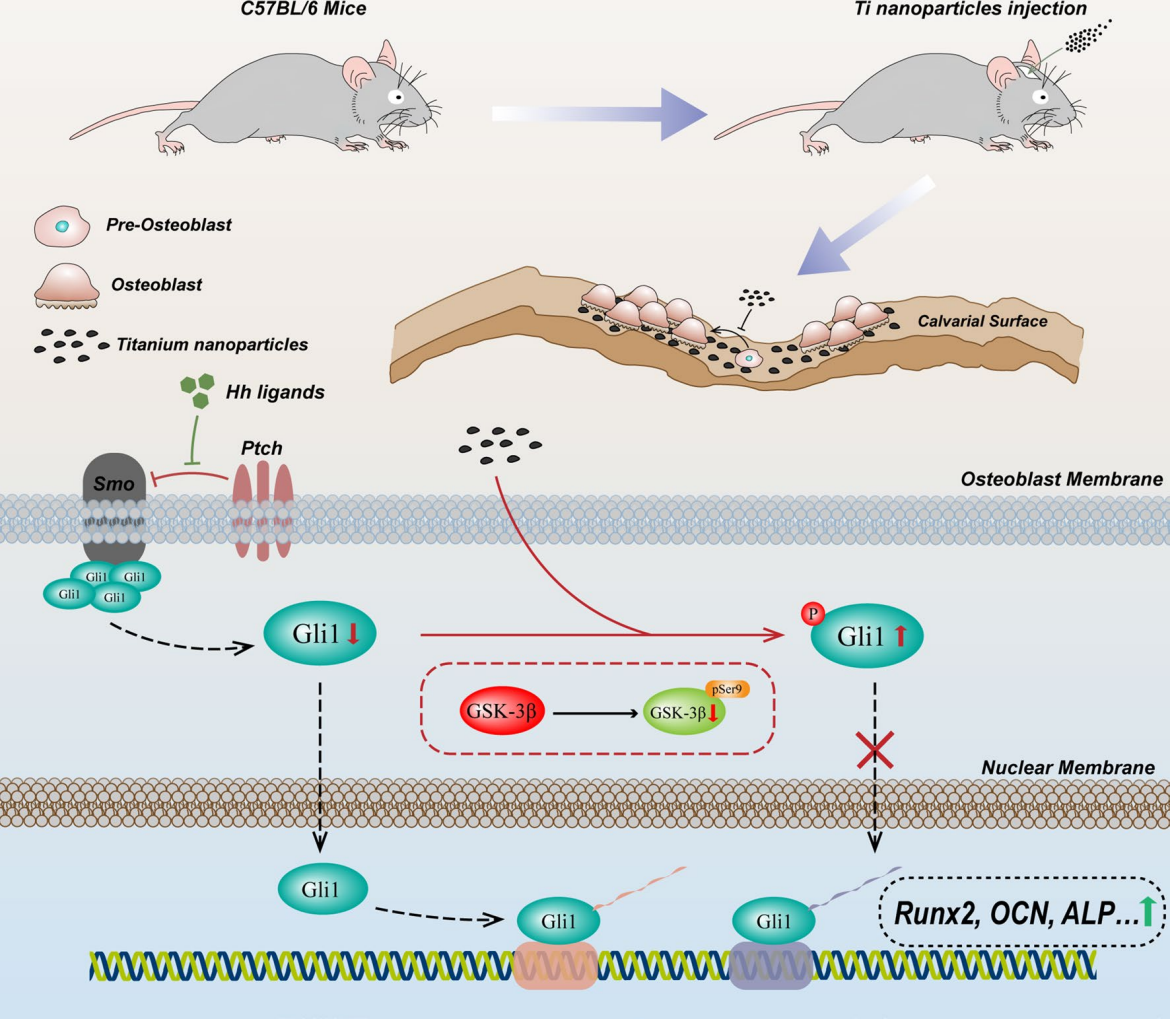

**Supplementary Information:**

The online version contains supplementary material available at 10.1186/s12951-022-01351-7.

## Introduction

Periprosthetic osteolysis (PPO) and ensuing aseptic loosening often contribute to prosthetic instability and surgical failure after total joint arthroplasty (TJA), which are common and potentially devastating long-term complications [1]. At present, revision surgery is unanimously thought to be the essential treatment for a failed prosthesis. Meanwhile, in the USA, it is projected that by 2030, the demand for revision of total knee arthroplasty (TKA) and total hip arthroplasty (THA) surgery will grow by over 600% and 130% from the corresponding levels in 2005, respectively [2]. Due to the high costs of revision surgery, its poor postoperative clinical effects, and the short prosthesis survival duration, revision surgery does not ultimately solve these complications [3]. Therefore, the need to explore the in-depth pathological mechanism of PPO to further develop new ideas for the prevention and treatment of PPO is urgent.

Although the mechanism by which PPO occurs is still indistinct, it is widely established that implant-derived nanoparticles, such as titanium (Ti), chromium (Cr), polyethylene and ceramic shed, produced by friction movement of artificial joints are the main culprit and can strongly induce a series of biological reactions, including the aggregation of immune cells and secretion of excessive inflammatory cytokines, and disrupt the balance between osteoblastic bone regeneration and osteoclastic bone resorption [4, 5]. Specifically, in the presence of wear particles, a variety of proinflammatory chemokines and cytokines was massively secreted by local active immune cells such as macrophage and T lymphocytes. These cytokines can not only in turn recruit osteoclast precursors, but also promote osteoclast differentiation and activation through stimulating stromal cells to secret plenty of receptor activators of nuclear factor-κB ligand (RANKL) [6]. Therefore, massive abnormal activated osteoclasts aggregate at the prosthesis interface, which eventually leads to abnormal bone resorption and implant loosening. Despite the critical roles of inflammation and osteoclast activation in particle-induced osteolysis, the inhibition of inflammation and/or osteoclastic activity alone cannot effectively rescue particle-induced osteolysis at the implant interface [7–9], indicating that other essential factors may be involved in particle-induced interface bone loss. In fact, the balance of bone turnover in the implant interface is dictated by not only bone resorption but also bone formation [10, 11]. Recent studies targeting the improved osseointegration of implants by recruitment of bone marrow-mesenchymal stem cells (BM-MSCs) and rescue of osteogenic capacity have made remarkable progress, which indicates that osteoblasts exert an important effect in osseointegration and maintaining bone mass around the implant [12, 13]. Meanwhile, some studies have explored the effect of osteoblasts under particle intervention and preliminarily demonstrated the negative effect of wear particles on osteogenic capacity [14, 15]. Therefore, the inhibition of osteogenesis caused by wear particles synergistically dominates bone loss at the implant interface. However, research on the in-depth mechanism of osteogenesis inhibition remains limited. Thus, exploring the underlying mechanism by which osteogenic capacity is inhibited under conditions of particle intervention is eagerly anticipated.

The Hh pathway is an evolutionarily conserved signaling pathway which participates in many fundamental processes in vertebrates, such as cell proliferation and differentiation, as well as skeletal development and homeostasis [16, 17]. Consistent with the essential role of the Hh pathway in development, the disorder of Hh pathway has been implicated in many human disorders, including cancer and skeletal disease [18, 19]. In mammals, the Hh pathway is composed of three Hh ligands [Sonic hedgehog (Shh), Desert hedgehog (Dhh) and Indian hedgehog (Ihh)], two transmembrane protein receptors [Patched receptor (Path) and Smoothened receptor (Smo)], and the five-zinc finger transcription factors [20]. Gli, which functions as a major downstream transcription factor of the Hh signaling pathway, affects gene transcription upon translocation from the cytoplasm to the nucleus. Notably, this process is strictly regulated by a complicated mechanism involving the phosphorylation and proteasome degradation of Gli factors [21–23]. Present studies revealed the key role of Hh signaling pathway in osteoblast differentiation and bone formation [24, 25]. Specifically, Hh signaling pathway activation was proved to participate in differentiation of MSCs to osteoblasts and chondrocytes [26, 27]. In addition, studies showed that activation of the Hh-Gli1 signaling pathway can promote bone mass by upregulating osteotropic cytokines such as Runx2 [28]. Of interest, a recent study on an apatite-binding nanoparticulate agonist of hedgehog signaling establishes a novel biomaterial design to specifically improve osteogenesis in bone defects by promoting Hh signaling [29]. Given the encouraging role of Hh-Gli1 signaling in osteogenesis, it has aroused our great interest whether the Hh signaling pathway is involved in osteogenesis inhibition in particle-induced osteolysis. However, to the best of our knowledge, whether Hh-Gli1 signaling contributes to the pathological features of wear particle-induced osteoblast remains unclear.

**Scheme 1:**
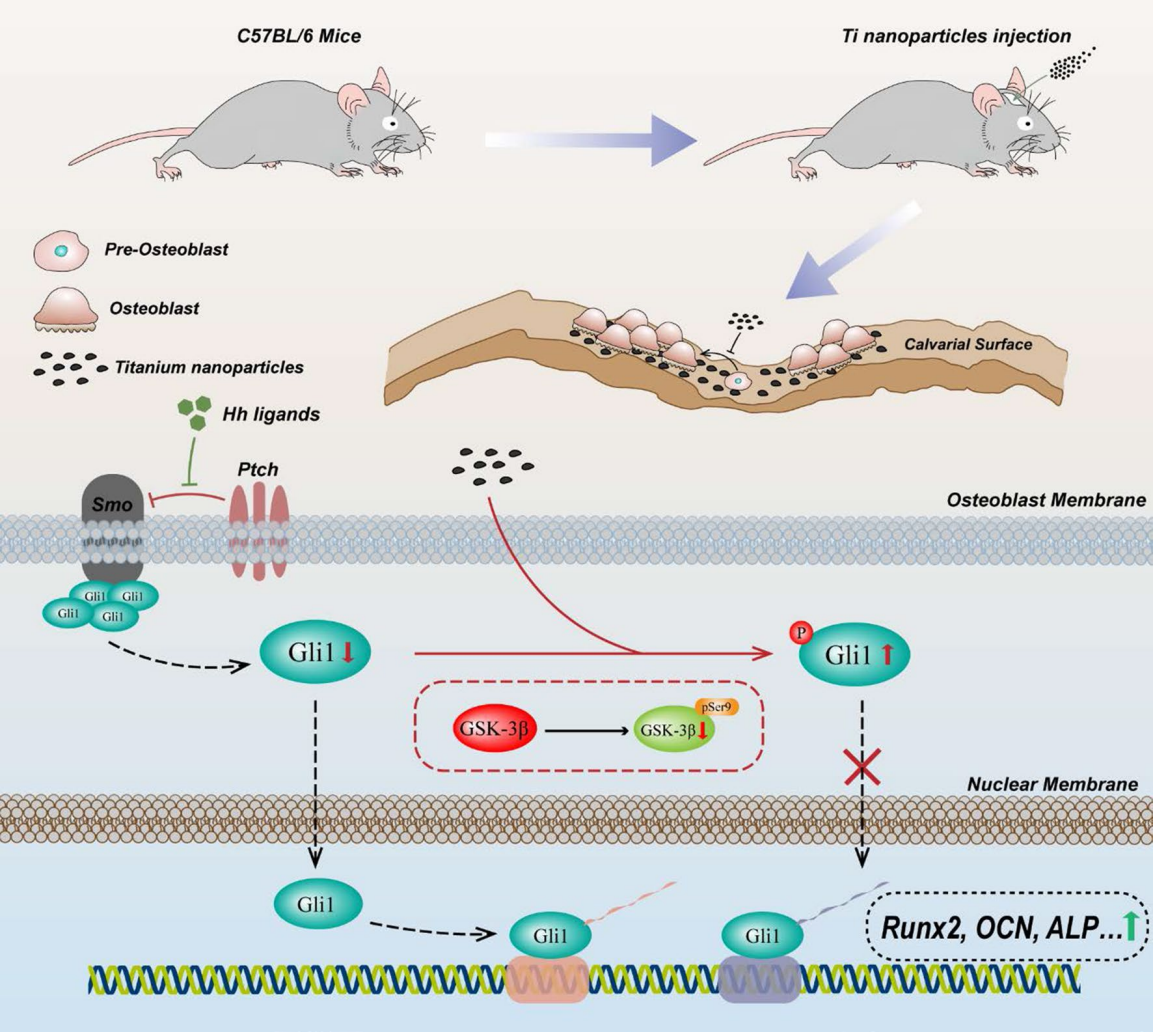
Schematic illustration showing the inhibition mechanism of osteogenesis in wear particle-induced osteolysis. Abnormal activation of glycogen synthesis kinase 3β (GSK-3β) triggered by Ti nanoparticles participates in the posttranslational modification and degradation of Gli1, which is the key transcription factor of osteogenesis in the Hedgehog pathway. In addition, inactivation of GSK-3β rescued Ti nanoparticle-induced osteogenesis inhibition as a result of increasing the accumulation of Gli1 and its translocation from the cytoplasm to the nucleus

Given these studies, we hypothesized that Ti nanoparticle-induced osteolysis following implantation of the prosthesis was tightly related to osteogenesis inhibition, and mechanistically, such inhibition is caused by a direct impairment of Hh-Gli1 signaling. To test this hypothesis, we explored the pathological features of Ti nanoparticle-induced osteoblasts in vitro and in vivo. Importantly, the underlying mechanism involved negative regulation of GSK-3β in the Hh-Gli1 signaling was first elucidated in Ti nanoparticle-induced inhibition of osteogenesis. Consistently, a Ti nanoparticle-induced calvarial osteolysis model further verified this cascade mechanism in vivo. Taken together, our results indicated that GSK3β-mediated impairment of the Hh-Gli1 signaling indeed contributed to Ti nanoparticle-induced osteogenesis inhibition and provided a basis for the inhibition of GSK-3β as a potential treatment strategy in wear particle-induced osteolysis (Scheme 1).

## Methods and materials

### Materials

Ti nanoparticles were purchased from Nanjing Emperor Nano Materials Company. The nanoparticles were first stored at 300 °C for 12 h. Then, it was soaked in 75% ethanol for 2 days to remove the residuary endotoxins. Ti nanoparticles characteristics were assessed through a scanning electron microscope (SEM). Frequency distribution of diameters was analyzed by ImageJ. TWS119 (Selleck Chemicals, Houston, USA) and GANT58 (APExBIO Technology LLC, Houston, USA) were dissolved in dimethyl sulfoxide (DMSO, Sigma) at a concentration of 100 mM and 40 mM, respectively, and stored at − 20 °C.

### Cell culture and osteoblast differentiation

A cell culture of preosteoblastic mouse MC3T3-E1 cells (FUHENG Biotechnology Co., Ltd, Shanghai, China) were conducted in Dulbecco’s modified Eagle’s medium (DMEM, Gibco) containing 10% fetal bovine serum (FBS, Shanghai XP Biomed Ltd.) as well as 1% penicillin and streptomycin (Gibco). The cells were grown at 37 °C in an incubator containing 5% CO_2_. For osteogenic differentiation, the cells were cultured in different well plates with DMEM supplemented with 0.5 mM vitamin C, 0.1 μM dexamethasone and 10 mM β-glycerophosphate. The culture medium was replenished every 3 days. TWS119 and GANT58 were pretreated to investigate the signaling mechanism. Ti nanoparticles were added at a proper concentration, which is similar to the concentration of wear particles retrieved from the tissue surrounding prosthetics [30, 31].

### Cell proliferation and viability assay

The cytotoxicity of Ti nanoparticles, TWS119 and GANT58 to MC3T3-E1 cells was assessed by cell counting Kit-8 (CCK-8; Beyotime) according to the manufacturer’s protocol. MC3T3-E1 cells were added to 96-well plates (8000 cells per well), and followed by Ti particles intervention at distinct densities and for different days of culture. TWS119 and GANT58 were added and cultivated for 3 days. Subsequently, the culture medium was replaced and incubated with DMEM containing a 10% volume of CCK-8 solution again for 1 h at 37 °C. Lastly, the absorbance was measured at a wavelength of 450 nm.

### Real-time PCR analysis

In accordance with the manufacturer’s instructions, total RNA from different samples was extracted using TRIzol reagent (Beyotime). The concentration of RNA was measured through a NanoDrop 2000 spectrophotometer (Thermo Fisher Scientific). Reverse transcription was performed to synthesize complementary DNA (cDNA) using 1 μg of isolated RNA according to the manufacturer’s protocol. To quantify expression of target genes, synthesized cDNA was used as templates for real-time PCR and each cDNA sample was mixed with qPCR Master Mix and primers listed in Additional file 1: Table S1. Real-time PCR was performed in a CFX96™ thermal cycler (Bio-Rad Laboratories) with eight tubes (NEST Biotechnology) that allowed amplification reactions. Every sample detection was performed in triplicate, and the fold change was calculated based on the comparative 2^−ΔΔCq^ method according to a previously described protocol [32].

### Western blot analysis

After different samples were harvested, cells were firstly washed twice using PBS and lysed at 4 °C for 30 min with radioimmunoprecipitation assay (RIPA; Beyotime) buffer. Then, following centrifuging the lysates at 12,000 rpm for 30 min at 4 °C, the supernatant fractions were collected. As directed by the manufacturer, the concentration of protein was determined by using a BCA kit (NCM Biotech, Soochow). A nuclear and cytoplasmic protein extraction kit (Beyotime) was applied to extract nuclear protein and cytoplasmic proteins according to the instructions provided by the manufacturer. After that, approximately 15 μg of nuclear/cytoplasmic or total cell proteins were resolved with sodium dodecyl sulfate-polyacrylamide gel electrophoresis (SDS-PAGE, Beyotime) and transferred to a polyvinylidene fluoride membrane (Bio-Rad Laboratories). After sealing with blocking buffer, the membrane was incubated in primary antibody solution, including antibodies against Runx2 (1:1000), OCN (1:1000), osterix (1:1000), pSer9-GSK-3β (1:000), GSK-3β (1:000), Gli1 (1:500), β-Actin (1:1000), β-tubulin (1:500), and Lamin-A (1:1000), at 4 °C for 12 h. Then the membrane was washed 5 times with western blot washing solution (Beyotime) before the secondary antibody solution was added at a dilution ratio of 1:5000 for 60 min. Finally, protein bands images were visualized using enhanced chemiluminescence (ECL, NCM Biotech, Soochow). The quantitation of protein grey level was achieved by Image Lab software Version 3.0 (Bio-Rad, USA).

### Alkaline phosphatase (ALP) staining

In brief, MC3T3-E1 cells were performed alkaline phosphatase (ALP) staining at the day 7. Briefly, cells were first fixed in 4% paraformaldehyde for 10 min. Then, cells were washed 3 times with cold PBS at 4 ℃. The BCIP/NBT (Beyotime) working solution was subsequently added and then incubated for 10 min. Images were captured by an inverted microscope (Zeiss). Absorbance was assessed at a wavelength of 520 nm (BioTek Instruments, Inc., USA) with an alkaline phosphatase quantification kit (Jiancheng Bioengineering Institute, Nanjing, China), in accordance with the manufacturer’s recommendations.

### Alizarin red S staining

The MC3T3-E1 cells were incubated in osteogenic medium for 21 days. Before staining, 4% paraformaldehyde was used to fix cells for 20 min. Incubation of cells was performed in an Alizarin red S (ARS) solution (Cyagen Biosciences) at pH 4.2 for 40 min. The images were taken after 3 times washing with ddH_2_O. After that, the calcium nodules were treated with 5% perchloric acid solution at 37 °C for 30 min, and the optical density was assessed at 420 nm.

### Cell immunofluorescence staining

MC3T3-E1 cells were seeded on coverslips in 24-well plates in DMEM for 12 h and then cultured in osteogenic differentiation medium with different interventions. Cells were seeded in 24-well plates at a density of 2 × 10^4^ per well. After osteogenic differentiation, all cells on coverslips were fixed with 4% paraformaldehyde for 20 min, followed by infiltration with 0.2% Triton X-100 (Beyotime) for 20 min. Blocking solution was then used to seal the cells for 60 min. Further, the primary antibodies were added and incubated at 4 °C for 12 h, including anti-OCN (1:200), anti-Gli1 (1:500) and anti-Runx2 (1:200) antibodies. Then, washing the cells 3 times by PBS and incubated in the secondary fluorescent antibody (Alexa Fluor^®^ 488) and TRITC Phalloidin (Yeasen) for 1 h. Coverslips were placed with fluorescence anti-fading solution containing DAPI (Beyotime) on microscope slides. The images were obtained by a fluorescence microscope (Zeiss). Nuclei were stained with DAPI (blue), Gli1, Runx2 and OCN-positive cells are depicted in green. The actin cytoskeleton was stained with phalloidin in red. Cell nuclei were identified using DAPI^+^ regions and then colocalized pixel-wise with green regions to assess the nucleus translocation level of Gli1. A ratio of integrated density to the area (IntDen/Area) was expressed as average fluorescence intensity using ImageJ.

### Animals and surgical procedure of the particle-implanted murine calvarial model

All experimental procedures in vivo were authorized by the Animal Ethics Committee of the First Affiliated Hospital of Soochow University (201905A116) and were performed according to the guidelines of the Care and Use of Laboratory Animals.

The well-established particle-implanted murine calvarial model was set up with 8-week-old male C57BL/6 mice. The details of the surgical operation are shown in Additional file 1: Fig. S1. 40 mice in total weighing an average of 20 ± 2 g were used and randomly separated into four groups (10 mice per group). All mice were anesthetized by intraperitoneal injection of 3% pentobarbital sodium (60 mg/kg) prior to the operation. After shaving, local disinfection was carried out with iodophor. After cutting the skin, the periosteum on the surface of the calvarias was scraped out. Then, 40 μl sterile phosphate buffered saline (PBS) containing 40 mg Ti nanoparticles was injected onto the calvaria surface. The sham group was injected with 40 μl PBS only. Finally, sew and close the incision. Mice received a daily intraperitoneal injection of TWS119 at a dose (10 mg/kg/day) in the TWS119 treatment group, TWS119 cotreated with GANT58 at a dose (20 mg/kg/day) in the TWS119 + GANT58 group and only equal volume of PBS and DMOS mixture in sham and vehicle group. In brief, 40 mice were randomly assigned to four groups (n = 10 in each group): sham operation group, vehicle group, TWS119 and TWS119 + GANT58 treatment group. All mice were euthanized on day 14 following the operation and all calvaria were then harvested.

### Micro-CT analysis

The calvaria were assessed by high-resolution micro-CT (SkyScan1176, Belgium) at 9 μm, 50 kV, and 200 μA. Before assessments, most of the Ti nanoparticles attached to the calvaria surface were gently scraped off using a soft gauze in order to avoid metal artifacts in following analysis. A region of interest exposed to Ti particles which located near the sagittal suture was analyzed and three-dimensional (3D) images rebuilding was conducted. The reconstruction program in SkyScan was applied to evaluate the morphometric parameters (bone volume per total volume (BV/TV, %), bone surface per bone volume (BS/BV, 1/mm), trabecular number (Tb. N, 1/mm), and total porosity (%)).

### Histological staining and analysis

All the calvaria were fixed in 10% formalin for a minimum of 2 days. After assessment by high-resolution micro-CT, the calvaria were decalcified with 10% ethylenediaminetetraacetic acid (EDTA, Sigma) for 28 days, embedded and sectioned into 6 μm sections. Lung, liver and kidney tissues from the four groups were harvested immediately after the mice were sacrificed and then fixed in 10% formalin. Next, they were embedded and sliced into 6 μm sections. H&E staining was performed to investigate the morphological changes of tissues following the manufacturer’s protocols.

A Masson trichrome staining kit (Leagene Biotech. Co., Ltd, Beijing) was used following the manufacturer’s protocols to evaluate the osteogenesis of new immature collagen by detecting the blue area after coloration of the tissues. The new immature collagen in the blue zone implied new bone formation. Morphological analysis of new immature collagen was performed through quantifying the blue staining average area percentage (average%) of five fields in each histopathologic section. The pictures were captured through Axio-Cam HRC microscope (Carl Zeiss, Germany).

### Statistical analysis

All data were represented as a mean ± standard deviation and analyzed with GraphPad Prism 7.0 software (GraphPad Software, Inc., USA). The Levene test was used to examine the equality of variance. One-way ANOVA was utilized to determine statistical significance for multiple comparisons, and Student’s t-test was performed to verify the significance of differences between two groups. Differences were considered statistically significant at p < 0.05.

## Results

### Ti particles remarkably inhibited osteogenic capacity

First, to mimic the effect of Ti particles produced by implant materials on osteogenic capacity, Ti nanoparticles were chosen in this study. Firstly, the characteristics of the Ti nanoparticles morphology was observed by scanning electron microscopy (SEM, Additional file 1: Fig. S2A, B). Most Ti particles had irregular morphology, almost 80% of the particles ranged from 60 to 120 nm with a mean diameter of 90.32 ± 37.32 nm (Additional file 1: Fig. S2C) which were consistent with previous studies [33, 34]. The results of CCK-8 and inhibition rate analysis showed that the Ti particles were not cytotoxic and inhibition effect when applied at a concentration below 40 μg/cm^2^ Ti particles over 1, 3 or 5 days (Additional file 1: Fig. S3A–F). Hence, to investigate the effect of Ti particles on osteogenic differentiation in vitro, MC3T3-E1 cells were treated with 2.5 µg/cm^2^ and 5 µg/cm^2^ Ti nanoparticles to generate the low- and high-dose groups, respectively.

**Fig. 1:**
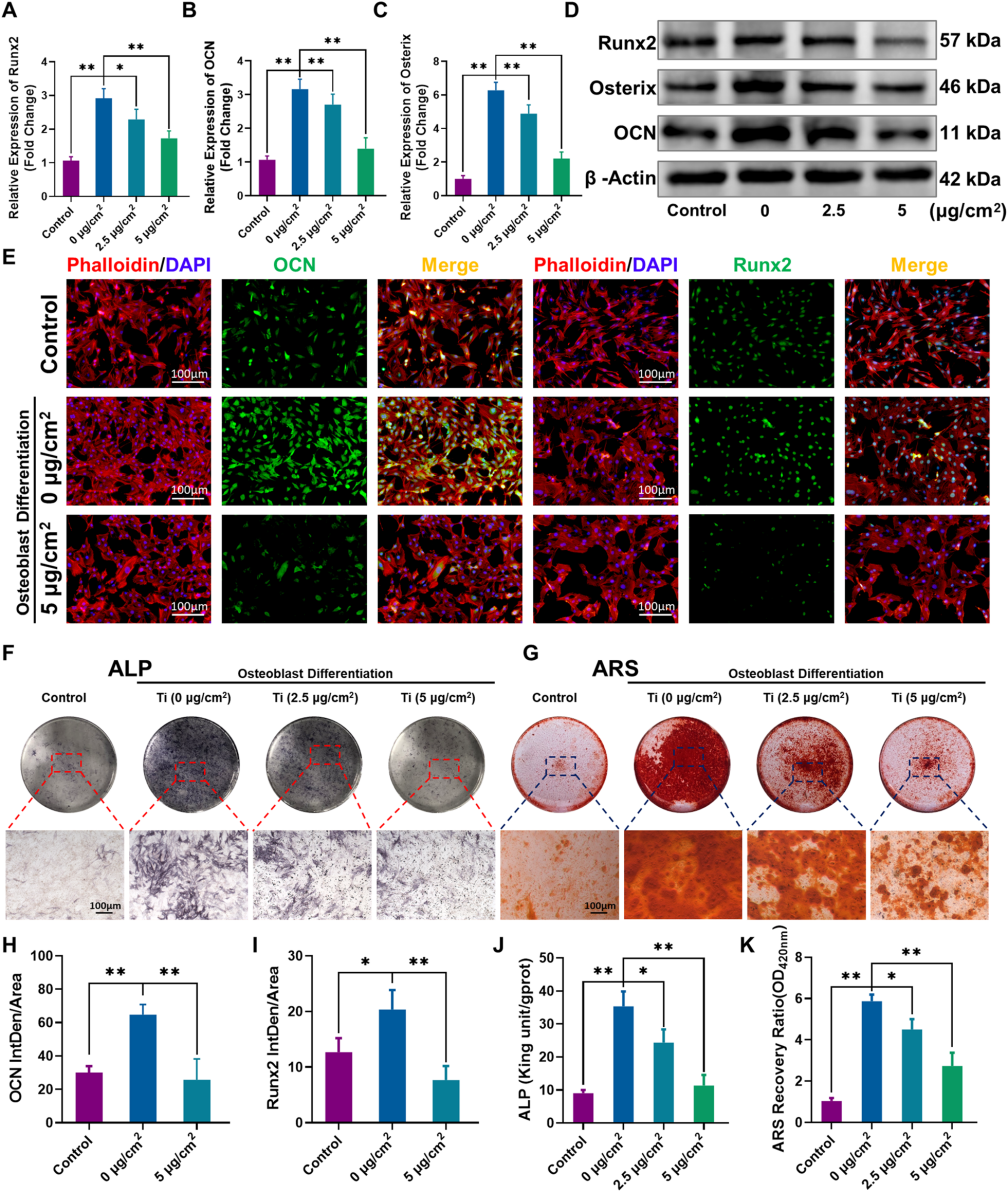
Ti particles remarkably inhibited the osteogenic capacity of MC3T3-E1 cells. **A**–**C** Runx2, OCN and Osterix mRNA levels. **D** Protein levels of Runx2, Osterix and OCN. **E** Representative images of immunofluorescence staining; red (phalloidin), green (OCN and Runx2), and blue (nuclei). **F**, **G** Representative images of ALP and ARS staining. **H**, **I** Quantitative analysis of the average fluorescence intensity of OCN and Runx2. **J**, **K** Quantitative analysis of ALP activity and ARS recovery ratio. All groups except the control group were cultured in osteogenic medium. n = 3 per group. *p < 0.05, **p < 0.01

The MC3T3-E1 cells were cultured in osteogenic medium with (2.5 µg/cm^2^ and 5 µg/cm^2^) or without (0 µg/cm^2^) Ti particle treatment. qRT-PCR analysis (Fig. 1A–C) demonstrated that the transcript levels of the osteogenic markers Runx2, OCN and Osterix were significantly upregulated in the 0 µg/cm^2^ group (osteogenic induction alone) compared to the control group. However, transcription of these markers was downregulated by Ti particle treatment in both 2.5 µg/cm^2^ and 5 µg/cm^2^ groups compared to the 0 µg/cm^2^ group, especially the 5 µg/cm^2^ group. Furthermore, the results of western blot and quantitative analysis were also consistent with the qRT-PCR results (Fig. 1D and Additional file 1: Fig. S4A–C). In addition, immunofluorescence staining was further performed to verify these changes. As shown in Fig. 1E, the expression of the secreted protein OCN and the nuclear transcription factor Runx2 was remarkably increased in the 0 µg/cm^2^ group, and Ti (5 µg/cm^2^) treatment significantly decreased the fluorescence intensity of Runx2 and OCN. Quantitative data of the average fluorescence intensity were measured in Fig. 1H and I and further indicated these effects. Our findings suggest that the Ti particles inhibited osteogenic differentiation marker transcription and protein expression in a dose-dependent manner.

Next, we assessed ALP activity and bone mineralization capacity of MC3T3-E1 cells upon Ti particles intervention at different doses. Cells were cultured in osteogenic medium with or without Ti particle treatment for 7 days and 21 days and separately subjected to the ALP assay and ARS. The ALP staining results showed that treatment with low- and high-dose Ti particles strongly inhibited the ALP activity of MC3T3-E1 cells (Fig. 1F). Quantitative analysis indicated that the ALP activity was remarkably decreased by approximately 31.42% and 68.57% in the 2.5 µg/cm^2^ and 5 µg/cm^2^ groups, respectively, compared to the 0 µg/cm^2^ group (Fig. 1J). Moreover, the ARS staining analysis confirmed the inhibitory effect of the Ti particles on bone mineralization capacity (Fig. 1G). Consistent with the ARS images results, semiquantitative analysis of the ARS results showed that the absorbance was greatly decreased by approximately 23.2% and 53.9% in the 2.5 µg/cm^2^ and 5 µg/cm^2^ groups, respectively, compared to the 0 µg/cm^2^ group (Fig. 1K). These results suggest that the Ti particles inhibited the osteogenic differentiation and mineralization capacity of the MC3T3-E1 cells in a dose-dependent manner.

### The Hh-Gli1 signaling cascade was inactivated by Ti particle treatment

**Fig. 2:**
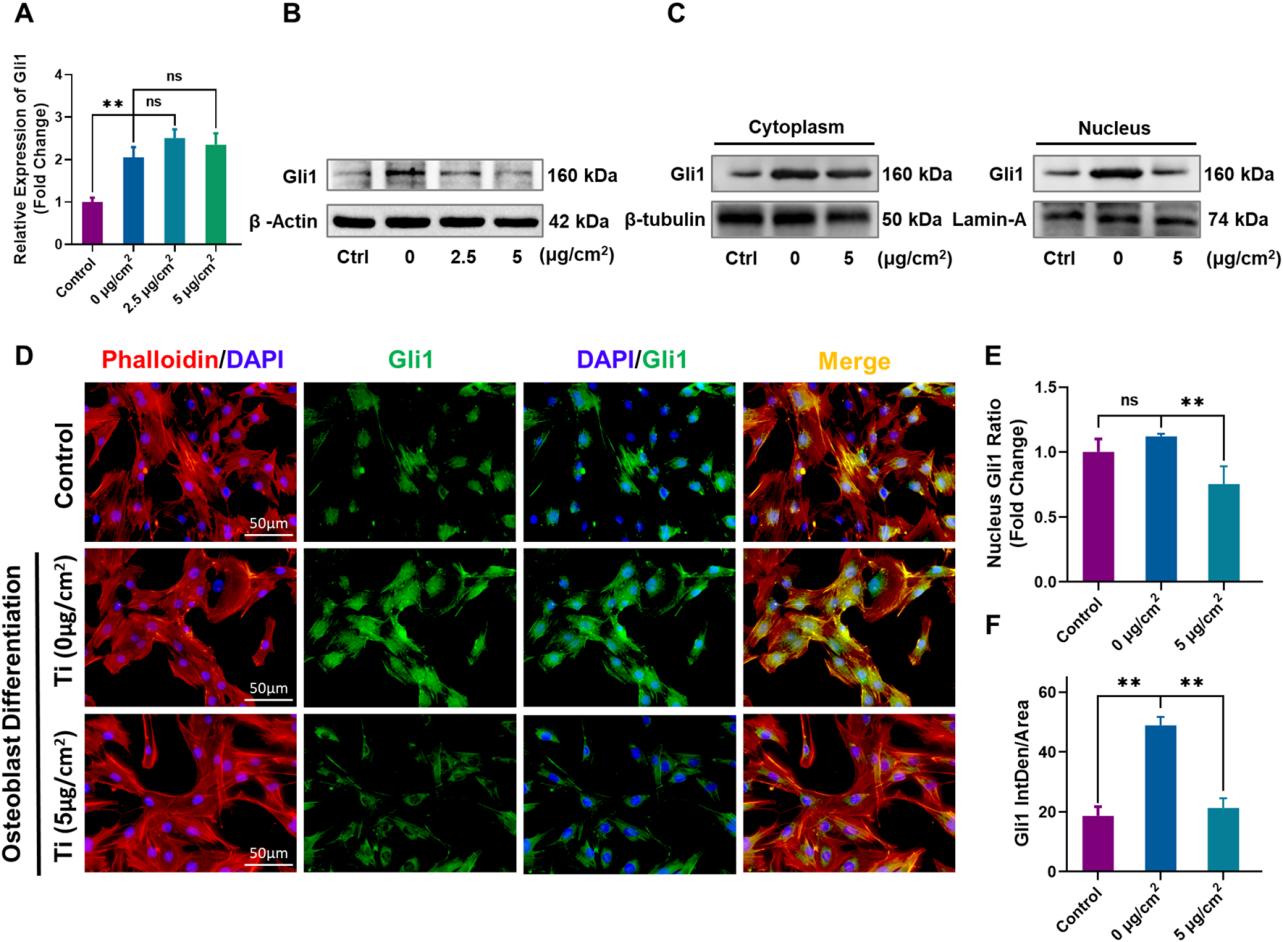
Ti particle intervention suppressed Hh-Gli1 signaling in MC3T3-E1 cells. **A** Gli1 mRNA levels. **B** Protein levels of Gli1 after intervention. **C** Protein levels of Gli1 in the cytoplasm and nucleus after intervention. **D** Representative images of immunofluorescence staining; red (phalloidin), green (Gli1), and blue (nuclei). **E** Quantitative analysis of nucleus Gli1 protein ratio. **F** Quantitative analysis of the average fluorescence intensity of Gli1. All groups except the control group were cultured in osteogenic medium. n = 3 per group. *p < 0.05, **p < 0.01, ns: no significance

In view of the crucial role of the Hh signaling pathway in embryonic development and the osteogenic differentiation of bone tissue, we first assessed the expression level of Gli1, which act as a crucial downstream transcription factor of the Hh signaling pathway. MC3T3-E1 cells were cultured in osteogenic medium with (2.5 µg/cm^2^ and 5 µg/cm^2^ groups) or without (0 µg/cm^2^ group) Ti particles. As shown in Fig. 2A, qRT-PCR analysis revealed that the mRNA level of Gli1 was remarkably upregulated in 0 µg/cm^2^ group compared to the control group. Meanwhile, the Gli1 mRNA level was also slightly upregulated in both the 2.5 µg/cm^2^ and 5 µg/cm^2^ groups compared to the 0 µg/cm^2^ group. However, there was no significant difference (p > 0.05). Then, we further investigated the protein expression of Gli1 under the same cell culture conditions by western blot analysis. In contrast to Gli1 mRNA expression level, Gli1 protein expression was significantly downregulated upon treatment with Ti particles at the two concentrations (Fig. 2B, p < 0.01). After the nuclear and cytoplasmic proteins were separated, western blot analysis was performed again. The results showed that Gli1 protein in both the cytoplasm and nucleus were obviously increased in the 0 µg/cm^2^ group compared to the control group. However, in the 5 µg/cm^2^ group, it was downregulated in both cytoplasm and nucleus, especially in the nucleus, compared to the 0 µg/cm^2^ group (Fig. 2C). Quantitative analysis of the relative grey levels further verified these changes (Additional file 1: Fig. S4D–F). More importantly, the ratio of the nuclear Gli1 protein levels was remarkably downregulated in the 5 µg/cm^2^ group compared to the 0 µg/cm^2^ group (Fig. 2E). In order to more intuitively evaluate the Gli1 nuclear translocation, cell immunofluorescence staining of Gli1 was performed as previous studies [35, 36]. The results indicated that Gli1 expression was obviously upregulated in the 0 µg/cm^2^ group in both cytoplasm and nucleus. However, 5 µg/cm^2^ Ti particle intervention significantly attenuated this change and especially decreased the expression level of Gli1 in the nucleus compared to that in the 0 µg/cm^2^ group (Fig. 2D). Quantitative analysis showed that the average fluorescence intensity of Gli1 was remarkably reduced in the 5 µg/cm^2^ group compared to the 0 µg/cm^2^ group (Fig. 2F). These results demonstrated that even though the Ti particles did not affect the transcription level of Gli1, they significantly reduced the protein level in both the cytoplasm and nucleus, especially in the nucleus. These results indicated that Ti particle treatment could trigger Gli1 protein degradation and nucleus translocation impairment at the posttranslational level.

### GSK-3β activation driven by Ti particles participated in degradation of the Gli1 protein

**Fig. 3:**
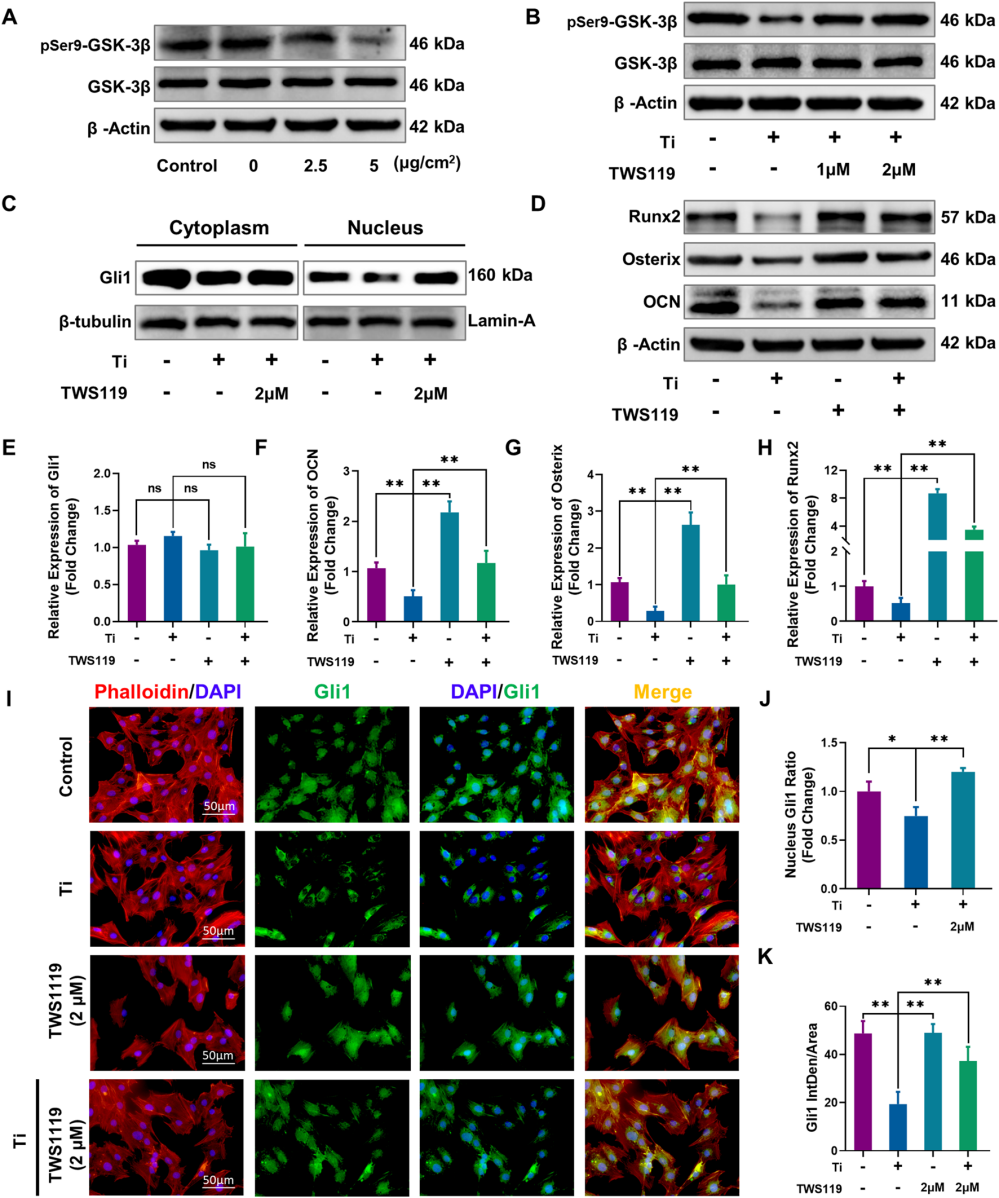
Blocking GSK-3β activates the Hh-Gli1 signaling pathway and rescues the Ti particle-induced inhibition of osteogenic differentiation. **A** Protein levels of pSer9-GSK-3β and GSK-3β, compared to 0 μg/cm^2^. **B** Protein levels of pSer9-GSK-3β and GSK-3β after TWS119 treatment. **C** Protein levels of Gli1 in the cytoplasm and nucleus after intervention. **D** Protein levels of Runx2, Osterix and OCN. **E**–**H** Gli1, OCN, Osterix and Runx2 mRNA levels. **I** Representative images of immunofluorescence staining; red (phalloidin), green (Gli1), and blue (nuclei). **J** Quantitative analysis of the nuclear Gli1 protein ratio. **K** Quantitative analysis of the average fluorescence intensity of Gli1. In **B**–**I**, the four groups were cultured in osteogenic medium, and the concentration of Ti particles was 5 µg/cm^2^. n = 3 per group. *p < 0.05, **p < 0.01, ns: no significance

In fact, the regulatory mechanism of the Hh signaling pathway is complex and remains unclear. Current studies have shown that Hh signaling may be regulated by several conserved negative regulatory kinases which can phosphorylate and inactivate Gli factors cooperatively [21, 22, 37]. GSK3-β is a serine/threonine protein kinase that has gained much attention for its role in a variety of signaling pathways such as Wnt/β-catenin as well as the Hh signaling pathway. Our previous study found that Ti particle-induced osteolysis is partly dependent on GSK-3β [38]. To investigate whether GSK-3β participates in the inactivation of the Hh signaling pathway, we first assessed the activity of GSK-3β under intervention by Ti particles. Through the western blot analysis, the GSK-3β protein level was found to be unaltered upon Ti particle treatment. However, the level of phosphorylated GSK-3β (Ser9) was significantly reduced in a dose-dependent manner (Fig. 3A). Quantitative analysis further verified these changes (Additional file 1: Fig. S4G and H). These effects eventually led to a decrease in the pSer9-GSK-3β/GSK-3β ratio in both the 2.5 µg/cm^2^ and 5 µg/cm^2^ groups compared to the 0 µg/cm^2^ group (Additional file 1: Fig. S4I), suggesting that GSK-3β was markedly activated by Ti particle stimulation.

To further determine whether GSK-3β was directly involved in degradation of the Gli1 protein driven by Ti particles during osteogenic differentiation of MC3T3-E1 cells, we next investigated Gli1 protein expression level upon GSK-3β inactivation via treatment with TWS119 (a selective GSK-3β inhibitor). First, analysis with the CCK-8 assay and inhibition rate revealed that treatment with TWS119 at concentrations under 2 µM had no toxicity or inhibition of cell proliferation (Additional file 1: Fig. S5A and B). Hence, treatment with 1 µM and 2 µM TWS119 was selected as the conditions for the low- and high-dose groups, respectively. The inhibitory effect of TWS119 on GSK-3β activity was first verified by western blot analysis (Fig. 3B). MC3T3-E1 cells were pretreated with or without TWS119 (a GSK-3β inhibitor, 1 µM and 2 µM) for 4 h and then cultured in osteogenic induction medium supplemented with 5 µg/cm^2^ Ti particles. Western blot analysis demonstrated that the pSer9-GSK-3β/GSK-3β ratio was significantly decreased in the Ti group, but this change was markedly reversed by treatment with TWS119 at both concentrations of 1 µM and 2 µM. Quantitative analysis further verified these changes (Additional file 1: Fig. S6A–C). Next, the Gli1 protein expression level upon GSK-3β inactivation was further investigated by western blot analysis (Fig. 3C). After the nuclear and cytoplasmic proteins had been separated, the results showed that 2 µM TWS119 treatment significantly increased Gli1 protein levels in both the cytoplasm and nucleus, especially in the nucleus, compared with those in the Ti group. Quantitative analysis of relative grey level and the nuclear Gli1 protein ratio further verified these changes (Additional file 1: Fig. S6D, E and Fig. 3J). As shown in Fig. 3I, immunofluorescence staining showed that Gli1 was strongly expressed and mainly localized in the nucleus after osteogenic differentiation in the control group. However, its expression level in the nucleus was mostly decreased upon Ti treatment, which indicated that the nuclear translocation of Gli1 was significantly inhibited. Treatment of TWS119 cells with or without Ti particles greatly increased the Gli1 expression level in both the cytoplasm and nucleus, especially in the nucleus, compared with that in the Ti groups. Quantitative analysis of the average fluorescence intensity of Gli1 further verified these changes (Fig. 3K). These results demonstrated that TWS119 could promote the accumulation and nuclear translocation of Gli1 by reducing degradation of the Gli1 protein driven by active GSK-3β.

### Activation of the Hh signaling pathway by GSK-3β inactivation strongly enhanced osteogenic differentiation and osteogenesis capacity upon Ti particle treatment

We next observed the capacity of MC3T3-E1 cells to undergo osteogenic differentiation following activation of the Hh signaling pathway by GSK-3β inactivation. qRT-PCR analysis showed that the mRNA expression of Gli1 was not significantly affected by Ti particles or TWS119 treatment (Fig. 3E). Ti treatment significantly decreased the mRNA levels of the osteogenic-related genes OCN, Osterix and Runx2. However, 2 μM of TWS119 treatment (TWS119 group) markedly increased the mRNA levels of these genes even cotreatment with 5 µg/cm^2^ Ti particles (Ti particles + TWS119 group), compared to their levels in the Ti group (Fig. 3F–H). Moreover, western blot analysis showed that 2 μM TWS119 treatment remarkably increased the expression of the osteogenic-related proteins OCN, Osterix and Runx2 even cotreatment with 5 µg/cm^2^ Ti particles, compared to their levels in the Ti group (Fig. 3D). Quantitative analysis of the relative grey levels further verified these changes (Additional file 1: Fig. S6F–H).

**Fig. 4:**
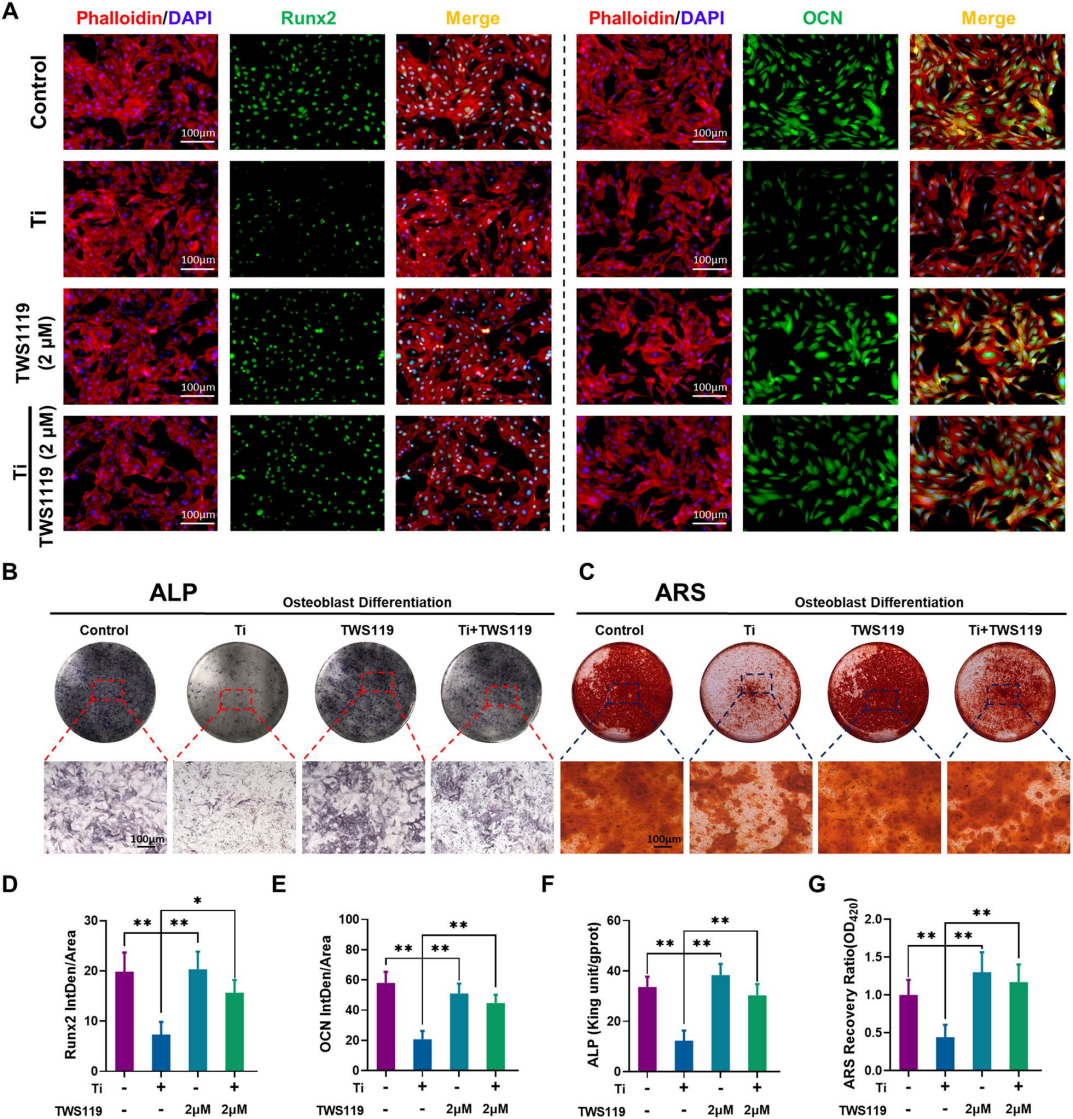
GSK-3β inactivation upregulates osteogenic differentiation and bone mineralization capacity. **A** Representative images of immunofluorescence staining; red (phalloidin), green (Runx2 and OCN), and blue (nuclei), n = 3 per group. **B**, **C** Representative images of ALP and ARS staining. **D**, **E** Quantitative analysis of the average fluorescence intensity of Runx2 and OCN. **F**, **G** Quantitative analysis of ALP activity and ARS recovery ratio. The four groups were cultured in osteogenic medium, and the concentration of Ti particles was 5 µg/cm^2^. *p < 0.05, **p < 0.01

As shown in Fig. 4A, the expression of Runx2 and OCN was further investigated by cell immunofluorescence staining upon activation of the Hh signaling pathway by GSK-3β inactivation. Ti (5 µg/cm^2^) treatment significantly decreased the average fluorescence intensity of Runx2 and OCN. However, TWS119 markedly upregulated Runx2 and OCN expression (TWS119 group) even cotreated with Ti particles (Ti + TWS119 group), compared to that in the Ti group. The results of quantitative analysis of the average fluorescence intensity were shown in Fig. 4D–E; these data further proved these evident effects. In addition, the results of ALP staining and ARS staining showed that treatment with TWS119 (TWS119 group) and even cotreated with Ti particles (Ti + TWS119 group) greatly increased ALP activity and bone mineralization capacity (Fig. 4B and C). The quantitative analysis of ALP activity and semiquantitative analysis of the ARS results further confirmed these changes (Fig. 4F and G). These results suggested that activation of the Hh signaling pathway by GSK-3β inactivation strongly enhanced osteogenic differentiation and osteogenesis capacity after Ti particle treatment.

### Hh-Gli1 signaling blockade attenuated the therapeutic effects of GSK-3β inactivation on osteoblast differentiation

**Fig. 5:**
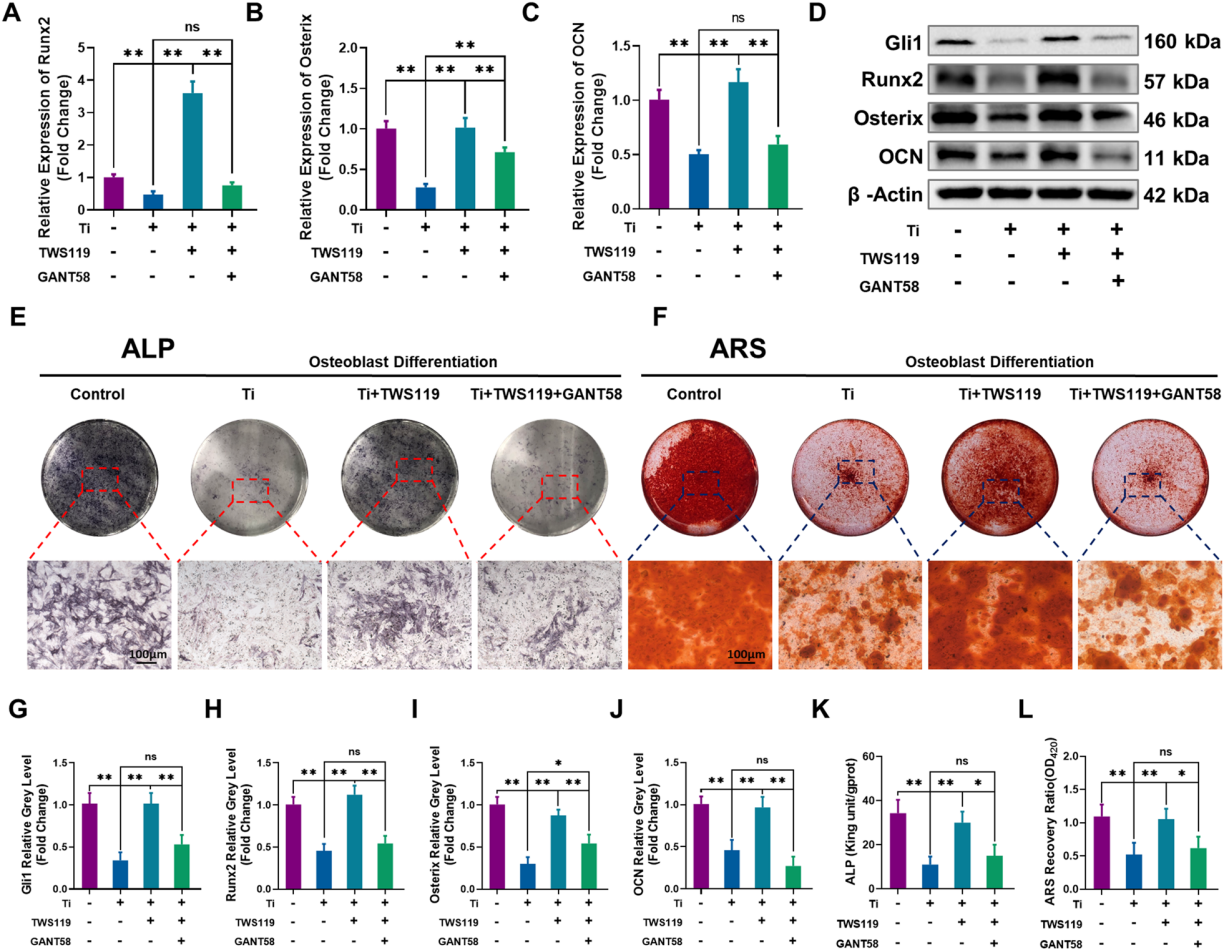
Hh-Gli1 signaling blockade attenuated the effects of GSK-3β inactivation on osteogenic differentiation. **A**–**C** Runx2, Osterix and OCN mRNA levels. **D** Protein levels of Gli1, Runx2, Osterix and OCN. **E**, **F** Representative images of ALP and ARS staining. **G**–**J** Quantitative analysis of the relative grey levels of Gli1, Runx2, Osterix and OCN. **K**, **L** Quantitative analysis of ALP activity and ARS recovery ratio. The four groups were cultured in osteogenic medium, and the concentration of Ti particles was 5 µg/cm^2^. n = 3 per group. *p < 0.05, **p < 0.01, ns: no significance

**Fig. 6:**
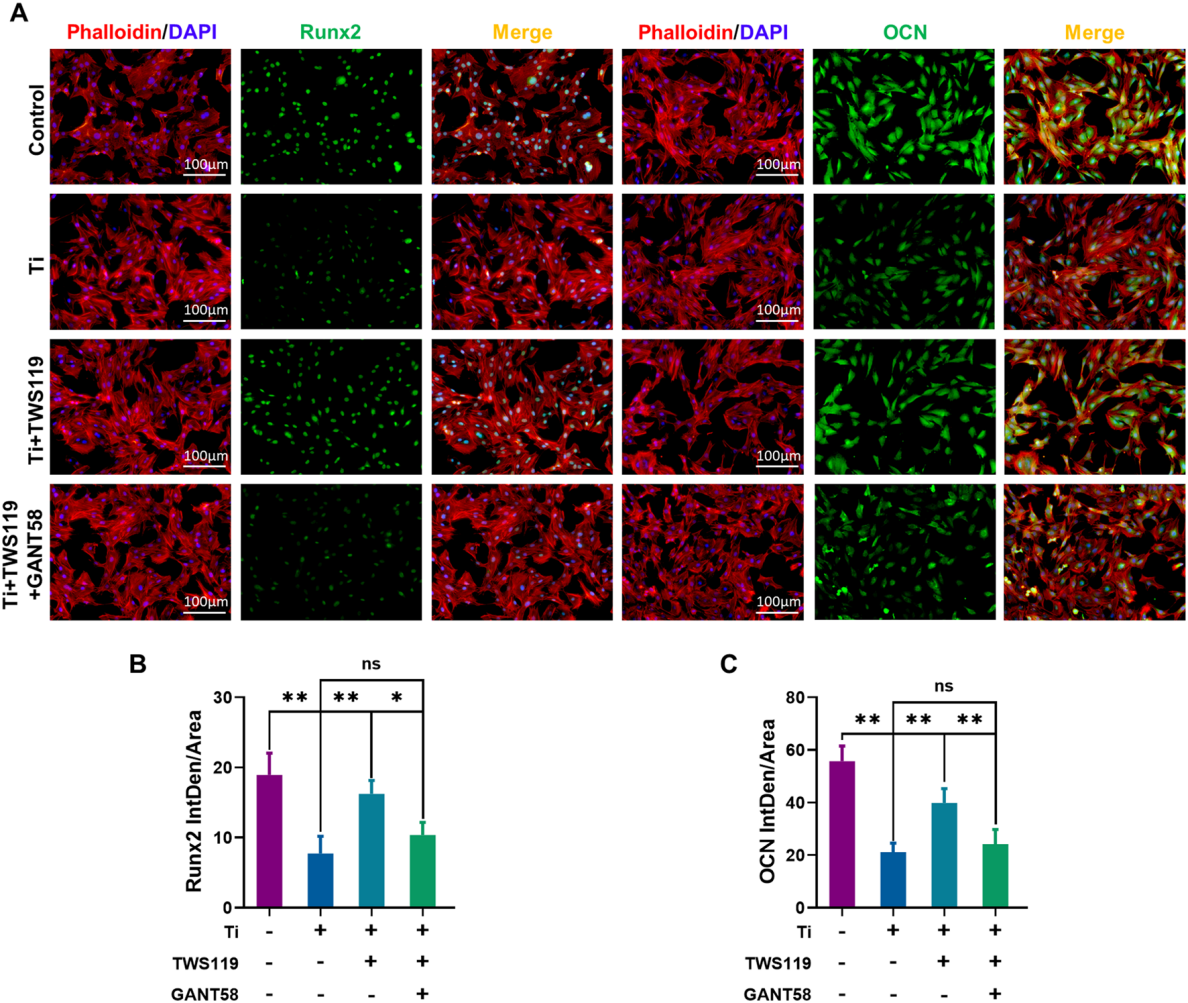
Hh-Gli1 signaling blockade reversed the upregulation effects of GSK-3β inactivation on Runx2 and OCN expression. **A** Representative images of immunofluorescence staining; red (phalloidin), green (Runx2 and OCN), and blue (nuclei). **B**, **C** Quantitative analysis of the average fluorescence intensity of Runx2 and OCN. The four groups were cultured in osteogenic medium, and the concentration of Ti particles was 5 µg/cm^2^. n = 3 per group. *p < 0.05, **p < 0.01, ns: no significance

To further verify the therapeutic mechanism of GSK-3β inactivation on Hh-Gli1 signaling pathway activation in Ti particle-induced osteogenic inhibition, MC3T3-E1 cells were co-cultured with GANT58 (a selective inhibitor of Gli1) to block Gli1 as previous studies [39, 40]. First, analysis of the CCK-8 assay and inhibition rate revealed that concentrations below 20 µM had no toxicity or inhibited cell proliferation (Additional file 1: Fig. S7A and B). Hence, 10 µM was selected as the concentration used for the GANT58-treated group. Next, the qRT-PCR analysis demonstrated that TWS119 significantly increased osteogenic-related gene transcription upon Ti particle intervention. However, the therapeutic effects of TWS119 were obviously reversed through treatment of exogenous GANT58 (Fig. 5A–C). Consistent with these findings, western blot analysis further verified that GANT58 impaired the positive effects of TWS119 on the protein expression of osteogenic genes compared to Ti + TWS119 group (Fig. 5D). Moreover, as shown in Fig. 5D, the protein expression of Gli1 was remarkable decreased in Ti group but obviously increased by TWS119 treatment. However, these changes were strongly blocked by exogenous GANT58. Quantitative analysis further verified these changes (Fig. 5G–J). In addition, the expression of OCN and Runx2 was further investigated by cell immunofluorescence staining (Fig. 6A). Ti particle (5 µg/cm^2^) treatment significantly decreased the fluorescence intensity of Runx2 and OCN. TWS119 markedly upregulated Runx2 and OCN expression upon Ti particle treatment compared to that in the Ti group. However, coculture with GANT58 significantly abrogated the protective effect of TWS119 compared to Ti + TWS119 group. The results of quantitative analysis of the average fluorescence intensity shown in Fig. 6B and C and further proved these effects. The results of ALP and ARS staining showed that treatment with TWS119 greatly enhanced ALP activity and the capacity of MC3T3-E1 cells undergoing bone mineralization (Fig. 5E and F). However, coculture with GANT58 significantly reversed the protective effect of TWS119 compared to Ti + TWS119 group. The results of quantitative analysis of ALP activity and semiquantitative analysis of the ARS results further confirmed these changes (Fig. 5K and L). Our findings revealed that the therapeutic effects of GSK-3β inactivation on cell differentiation were mediated by the Hh-Gli1 signaling pathway.

### The Hh-Gli1 signaling cascade is involved in a GSK3β-mediated mechanism in a murine calvarial osteolysis model

**Fig. 7:**
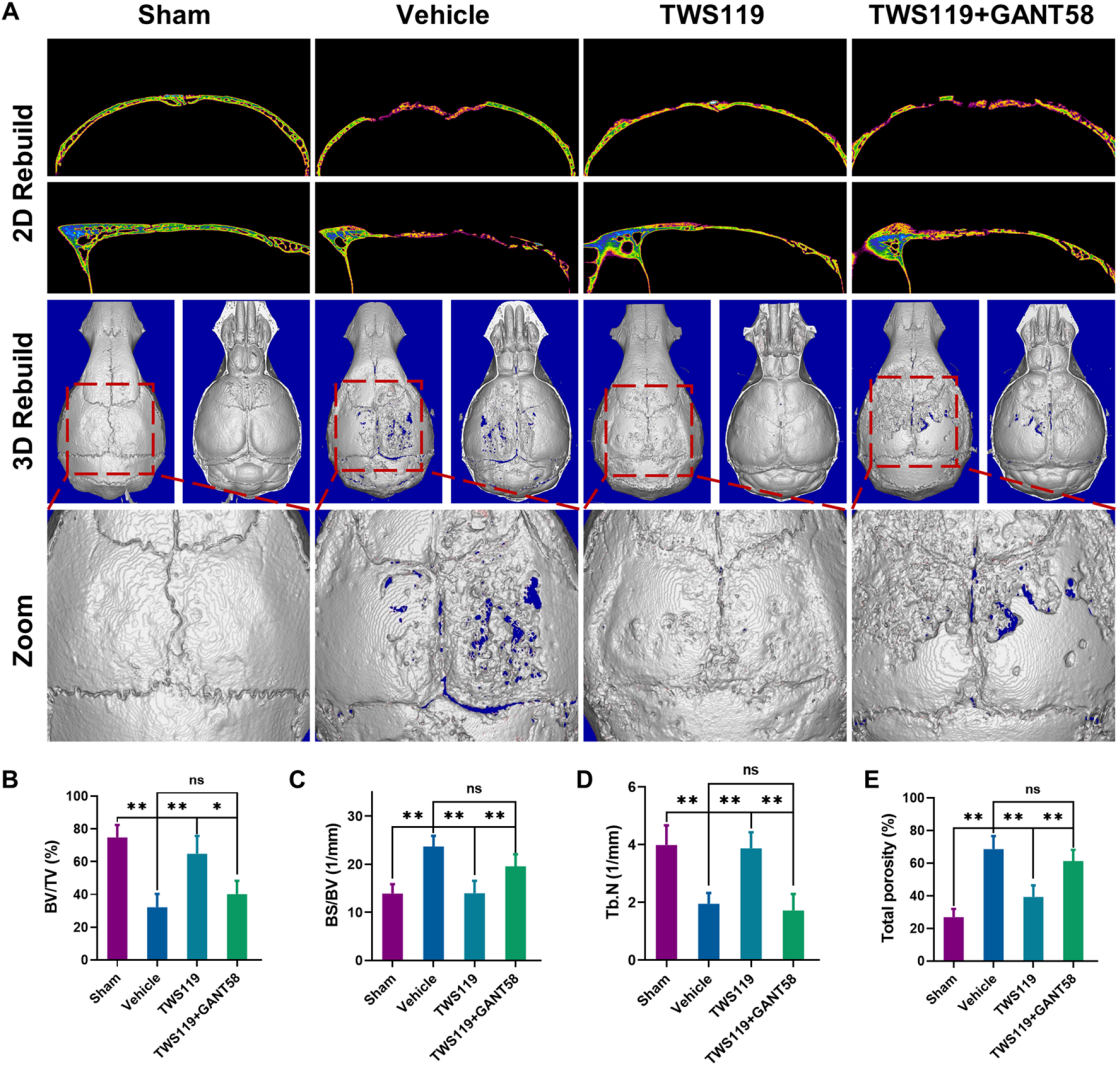
The Hh-Gli1 signaling cascade is involved in a GSK3β-mediated mechanism in vivo. **A** Representative images of 2D reconstruction in coronal and sagittal positions and 3D reconstruction. **B**–**E** Quantitative analysis of BV/TV (%), BS/BV (1/mm), Tb. N (1/mm) and total porosity (%). n = 6 per group. *p < 0.05, **p < 0.01, ns: no significance

In order to confirm the mechanism of bone loss involved GSK-3β-mediated Hh-Gli1 signaling pathway in vivo, a murine calvarial osteolysis model generated by Ti particle injection was performed to mimic the molecular pathogenesis in vivo. Micro-CT analysis was performed to assess the bone mass and degree of osteolysis within a region of interest (ROI) of calvaria exposed to Ti particles. The 2D and 3D reconstructed images showed that calvaria from the vehicle group was extensively eroded. Daily injection of TWS119 (10 mg/kg, i.p.) strongly alleviated particle-induced bone erosion. However, the protective effect of TWS119 was blocked by GANT58 (20 mg/kg, i.p.) treatment (Fig. 7A). More specifically, micro-CT analysis showed that TWS119 treatment significantly decreased bone destruction and loss, which was characteristic of the vehicle group. Meanwhile, changes in trabecular bone parameters, including the BV/TV, BS/BV, Tb. N and total porosity were also significantly abrogated in the TWS119-treated group compared to the vehicle group. However, these therapeutic effects were dramatically decreased in the GANT58 cotreatment group compared to TWS119-treated group (Fig. 7B–E). In addition, H&E staining of the lung, liver and kidney showed no toxicity in any of the groups of mice (Additional file 1: Fig. S8A). These findings suggested that inactivation of GSK-3β protected against Ti particle-induced bone destruction in a murine calvarial model via the Hh-Gli1 signaling cascade.

### Histological analysis of bone destruction and osteogenic capacity in vivo

**Fig. 8:**
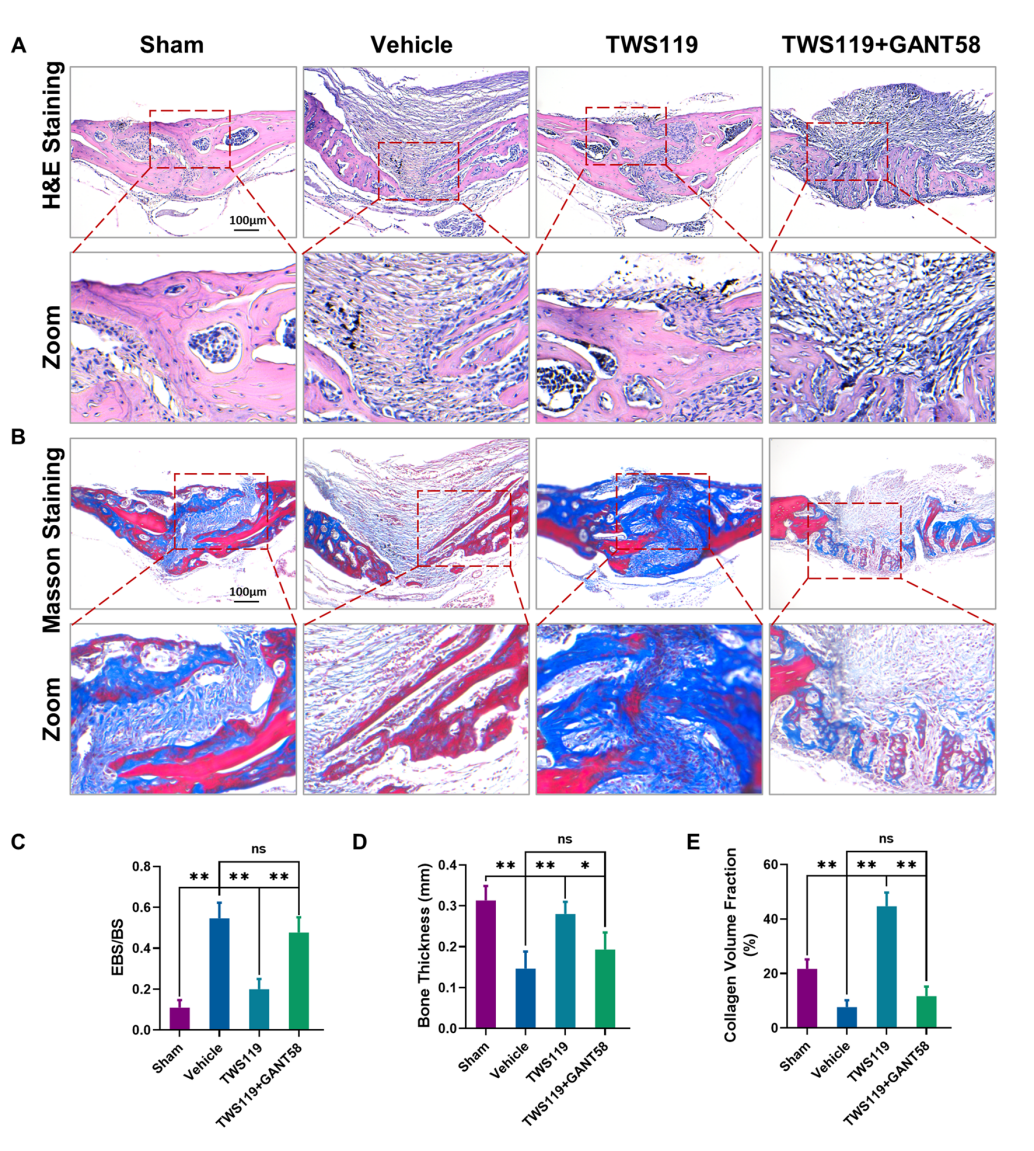
Histological analysis of bone destruction and osteogenic capacity in vivo. **A**, **B** Representative images of H&E and Masson trichrome staining. **C**–**E** Quantitative analysis of EBS/BS, bone thickness (mm) and collagen volume fraction (%). n = 6 per group. *p < 0.05, **p < 0.01, ns: no significance

To further explore the degree of bone erosion and osteogenic capacity in the different groups in vivo, histological H&E and Masson staining were performed. In particular, the Masson trichrome staining kit was used to evaluate the osteogenesis of new immature collagen by detecting the blue zone after coloration of the tissues. The new immature collagen in the blue zone indicated new bone formation. H&E staining showed extensive erosion and fibrosis proliferation on the calvarial surface in the vehicle group compared to the sham group. TWS119 treatment markedly alleviated this bone erosion and fibrosis. However, cotreatment with TWS119 and GANT58 had no significant protective effect against bone erosion and fibrosis induced by the Ti particles (Fig. 8A). Next, we further quantified the eroded bone surface to total bone surface (EBS/BS) ratio and bone thickness (BT). The results showed that the EBS/BS ratio remarkably increased in the vehicle group. However, this change was markedly rescued by treatment with TWS119. In contrast, there was no significant difference in the EBS/BS ratio between the vehicle and TWS119 and GANT58 cotreatment groups (Fig. 8C). In addition, quantitation of the BT value showed that Ti particle implantation significantly decreased the BT of the calvaria compared with that in the sham group. TWS119 treatment clearly rescued the decrease in BT compared to BT in the vehicle group. In contrast, cotreatment with TWS119 and GANT58 had no significant effect on BT compared to that in the vehicle group (Fig. 8D). Next, the results of Masson staining showed that the growth of new immature collagen fibers in murine calvaria was obviously decreased in the vehicle group, but TWS119 treatment strongly enhanced collagen fiber formation. However, no significant increase in collagen fiber formation was observed in the TWS119 and GANT58 cotreatment groups (Fig. 8B). Quantitative analysis of the collagen volume fraction further confirmed these changes (Fig. 8E). The results of these histological analysis demonstrated that GSK-3β inactivation by TWS119 protected murine calvaria from Ti particle-induced erosion and promoted osteogenic capacity partly via Hh-Gli1 signaling in vivo.

## Discussion

In recent years, surface engineering of titanium using nanotopography to regulate cell behavior has attracted many research attention. Many studies showed that the nano-scale modification of titanium surfaces such as nanotube structures have positive effect on osteogenesis and bone formation onto the implants through enhancing the adsorption of protein, osteogenic cell migration, angiogenesis [41–43]. More specifically, Yao Lin et.al investigated different sizes of titanium nanotubes on osteogenesis and impressively revealed that nanotube diameter of 70 nm provided an encouraging microenvironment for osteogenic differentiation which involved a mechanism of beta1-integrin/Hedgehog-Gli1 signaling activation [44, 45]. However, free nanoparticles generated owing to attrition of orthopedic implants surface demonstrated a reverse effect in local bone homeostasis instead. Recently, nanosized wear particles below 0.1 μm were found both in joint simulators and periprosthetic tissues [46, 47]. More and more studies revealed the key role of wear nanoparticles in osteolysis diseases [48, 49]. Studies also demonstrated that titanium nanoparticles do not only influence MSCs enzymes activity but also contribute to cytotoxic and genotoxic effects [50–52]. In addition, a study of intra-articular injection of nanoparticles revealed that the aggregated titanium nanoparticles deposited could result in oxidative damage in local join tissue [53]. Zhang et al. demonstrated that titanium nanoparticles can participate in inflammatory response and affect local bone homeostasis [34]. The present results indicated that the wear particles retained have negative potential effects on bone biological function.

Obviously, a series of biological reactions induced by wear particles in periprosthetic osteolysis have aroused extensive interest of scholars. Previous studies have extensively explored the underlying mechanisms of wear particles induced osteoclast activation. Targeting the abnormal bone resorption effects of osteoclasts, several active molecules, such as bisphosphonates and denosumab, which can act on osteoclast inhibition have been showed to be promising therapies on periprosthetic osteolysis. Nevertheless, it has been noted that long-term use of these medications comes with serious side effects, including atypical fractures of the femur and osteonecrosis of the jaw. Furthermore, considering that the maintenance of bone homeostasis relies on the dual regulation of osteoblasts and osteoclasts, the therapeutic efficacy against PPO of cures targeting only osteoclasts remains limited. In recent years, as the increasing attention paid to the role of osteoblast in periprosthetic osteolysis, scholars found that new biomaterial strategies targeting osteogenesis can significantly improve the bone integration capacity and prevented implant loosing [54, 55]. Wu et al. found that the blockade of wear particle-induced apoptosis and NF-κB activation in osteoblasts could clearly reduce osteolysis and increase the stability of the prosthesis [56]. In addition, our previous studies showed that relieving the inhibitory effect of Ti particles on osteoblasts could significantly alleviate particle-induced osteolysis [57, 58]. In view of alleviating the wear particle-induced reduction in periprosthetic osteogenesis is crucial for the prevention and treatment of PPO, it is necessary to further explore the molecular mechanisms underlying particle-induced osteogenic inhibition.

Hh signaling pathway in bone tissue during embryonic development and in maintaining bone homeostasis has received increasing attention in recent years. Previous studies showed that the Hh-Gli1 signaling pathway is crucial in mesenchymal progenitors, which are responsible for both normal bone formation and fracture repair [59]. Meanwhile, selective Hh signaling inhibition was shown to reduce the osteoblast differentiation and ectopic bone formation of human skeletal MSCs, indicating that targeting the Hh-Gli1 pathway may act as a therapeutic treatment in these diseases [60]. Lv et al. further summarized the important mechanistic role of Hh signaling in osteoblast development [25]. The pathological changes that occur in osteoblasts under particle intervention are complex and may involve a variety of signaling pathways. Therefore, considering the crucial role of the Hh signaling pathway in the proliferation and differentiation of osteoblasts, we are greatly interested in determining whether the Hh signaling pathway is involved in osteogenic inhibition upon intervention with wear particles.

As Gli proteins acted as a key transcription factor in Hh signaling and controls downstream target gene expression, the posttranslational modification and degradation of Gli proteins are crucial for the function of the Hh pathway. Studies have reported that full-length Gli (155) can be posttranslationally modified via negative kinases such as PKA, GSK-3β and CKI family members (CK1a and CK1e) and then cleaved to shorter forms (75). Gli-75 retains the zinc finger DNA-binding domain of Gli proteins but lacks the transactivation domain and nuclear export sequences. Thus, only unmodified full-length Gli proteins can accumulate in the nucleus as transcriptional activators. GSK-3β phosphorylation sites are required for the proteolysis of Gli proteins. The hyperphosphorylation of Gli proteins triggers ubiquitination and proteasome-mediated protein degradation [21, 37]. In our study, we focused on the negative regulatory effect of GSK-3β on the Hh-Gli1 signaling pathway. Studies have shown that the biological effects of GSK3-β are mainly exerted through its participation in the phosphorylation of substrates. Specifically, the phosphorylation state of the GSK-3β site can regulates the activity of GSK-3β, and GSK-3β activity could be negative regulated by phosphorylation at the N-terminal serine residue (Ser9) [61]. In addition, studies reported that activated GSK-3β could phosphorylate Gli1, and phosphorylated Gli1 was further degraded by the proteasomal degradation pathway [23, 62]. Considering the interaction between the negative effect of GSK-3β and the Hh-Gli1 signaling pathway in various cell types shown in previous studies, whether GSK-3β is involved in osteogenic inhibition by mediating the Hh-Gli1 signaling cascade in osteoblasts has received considerable attention.

Here, we investigated the role of Ti particles at different concentrations on the proliferation and osteoblast differentiation of MC3T3-E1 osteogenic precursor cells and then determined the mechanism of osteogenic inhibition caused by Ti nanoparticles in vitro and vivo. We revealed that Hh signaling was potently inhibited in particle-induced osteogenesis and that GSK-3β was potently activated in vitro. More importantly, GSK-3β inactivation by a selective inhibitor (TWS119), which promoted pSer9-GSK3β transformation, thus decreasing the GSK-3β/pSer9-GSK-3β ratio, greatly reversed Hh-Gli1 impairment, enhanced osteogenic capacity in vitro*,* and ameliorated Ti particle-induced bone destruction in a murine calvarial model. However, this therapeutic effect was blocked by treatment with a selective inhibitor of Gli1 (GANT58) in vitro and in vivo. In summary, we have ultimately concluded that Ti particles can inhibit osteogenesis through the posttranslational modification and degradation of members of the Hh-Gli1 signaling pathway and that the mechanism involves GSK-3β activation. Therapeutic strategies based on regulation of the Hh-Gli1 signaling cascade mediated by GSK3β are expected to inspire new ideas for the treatment of PPO.

In general, several limitations are existed in our study. Ti particles were used to mimic the wear particles produced by artificial joint prostheses; the friction interface was mainly formed of metal and polyethylene, and the produced particles were mainly polyethylene particles. However, given our proficiency in generating Ti particle-induced osteolysis models in vivo and in vitro and because Ti particles represent an important pathological factor in osteolysis, we used Ti particles to treat the vehicle group despite any potential limitations. In addition, the pathogenesis of Ti particle- and polyethylene particle-induced osteolysis are similar, the bone destruction resulted by these particles were both regulated via bone metabolism. Further studies using an osteolytic animal model that closely mimics the clinical features of PPO are warranted to confirm our findings. In addition, the in vitro findings showing that the Hh-Gli1 signaling cascade involves a GSK3β-mediated mechanism were not further validated in a murine calvarial model, except to validate the therapeutic effect of TWS119 and demonstrate that this therapeutic effect could be eliminated by selective inhibitors of Gli1 (GANT58). In our following studies, we will further carry out verification and examine the Hh-Gli1 signaling cascade in vivo in addition to clinical research focused on the Hh-Gli1 pathway and whether it can affect other cells involved in bone metabolism, such as bone immune cells and osteoclasts, through osteoblast-secreting factors or other methods.

## Conclusion

Comprehensively, our findings indicated that Hh-Gli1 signaling impairment exerts a crucial role in Ti particle-induced osteogenesis inhibition. The abnormal increase in GSK-3β activity mediated the inactivation and degradation of the Gli1 transcription factor, resulting in a decrease in the nuclear translocation level. Inactivation of GSK-3β kinase can enhance osteogenesis capacity through the Hh signaling pathway. These results could reveal a potential therapeutic strategy against wear particle-induced PPO.

## Supplementary Information

**Additional file 1: Figure S1. **(A) The detailed surgical steps of the particle-implanted murine calvaria model. **Figure S2.** (A, B) Representative Scanning electron microscopy (SEM) image of Ti nanoparticles. (C) Frequency distribution of Ti nanoparticles size. **Figure S3.** (A–C) Cell viability after incubation for 1, 3 and 5 days with different concentrations of Ti particles was assessed using a CCK-8 kit, *p < 0.05, compared with the 0 μg/cm^2^ group. (D–F) Cell inhibition rate after incubation for 1, 3 and 5 days with different concentrations of Ti particles. The data were expressed as the mean ± SD. **Figure S4.** (A–C) Quantitative analysis of the relative grey levels of Runx2, Osterix and OCN. (D–F) Quantitative analysis of the relative grey levels of Gli1. (G–I) Quantitative analysis of the relative grey levels of pSer9-GSK-3β and GSK-3β. All data were expressed as the mean ± SD. *p < 0.05, **p < 0.01, ns: no significance. **Figure S5. **(A) Cell viability after incubation for 3 days with different concentrations of TWS119 was assessed using a CCK-8 kit, *p < 0.05, compared with the 0 μM group. (B) Cell inhibition rate after incubation for 3 days with different concentrations of TWS119. The data were expressed as the mean ± SD. **Figure S6. **(A–C) Quantitative analysis of the relative grey levels of pSer9-GSK-3β and GSK-3β after TWS119 treatment. (D–E) Quantitative analysis of the relative grey levels of Gli1 after TWS119 treatment. (F–H) Quantitative analysis of the relative grey levels of Runx2, Osterix and OCN. All data were expressed as the mean ± SD. *p < 0.05, **p < 0.01, ns: no significance. **Figure S7.** (A) Cell viability after incubation for 3 days with different concentrations of GANT58 was assessed using a CCK-8 kit, *p < 0.05, compared with the 0 μM group. (B) Cell inhibition rate after incubation for 3 days with different concentrations of GANT58. **Figure S8.** (A) Representative images of H&E staining of lung, liver and kidney collected from the four groups. **Table S1.** Primer sequence.

## Abbreviations

BV/TV: Bone volume per total volume
BS/BV: Bone surface per bone volume
Tb. N: Trabecular number
BCIP/NBT: 5-Bromo-4-chloro-3-indolyl-phosphate/nitro blue tetrazolium
DMEM: Dulbecco’s Modified Eagle Medium
DMSO: Dimethyl sulfoxide
EDTA: Ethylene diamine tetraacetic acid
H&E: Hematoxylin and eosin
OCN: Osteocalcin
PPO: Peri-prosthetic osteolysis
qRT-PCR: Quantitative real-time polymerase chain reaction
Runx2: Runt-related transcription factor 2
TJA: Total joint arthroplasty
