# Supplementary material for: GSK-3β suppression upregulates Gli1 to alleviate osteogenesis inhibition in titanium nanoparticle-induced osteolysis
Source: J Nanobiotechnology. 2022 Mar 19;20:148. doi: 10.1186/s12951-022-01351-7 (PMC8934501; doi:10.1186/s12951-022-01351-7)
Supplement: Supplementary file 1 — Additional file 1: Figure S1. (A) The detailed surgical steps of the particle-implanted murine calvaria model. Figure S2. (A, B) Representative Scanning electron microscopy (SEM) image of Ti nanoparticles. (C) Frequency distribution of Ti nanoparticles size. Figure S3. (A–C) Cell viability after incubation for 1, 3 and 5 days with different concentrations of Ti particles was assessed using a CCK-8 kit, *p < 0.05, compared with the 0 μg/cm2 group. (D–F) Cell inhibition rate after incubation for 1, 3 and 5 days with different concentrations of Ti particles. The data were expressed as the mean ± SD. Figure S4. (A–C) Quantitative analysis of the relative grey levels of Runx2, Osterix and OCN. (D–F) Quantitative analysis of the relative grey levels of Gli1. (G–I) Quantitative analysis of the relative grey levels of pSer9-GSK-3β and GSK-3β. All data were expressed as the mean ± SD. *p < 0.05, **p < 0.01, ns: no significance. Figure S5. (A) Cell viability after incubation for 3 days with different concentrations of TWS119 was assessed using a CCK-8 kit, *p < 0.05, compared with the 0 μM group. (B) Cell inhibition rate after incubation for 3 days with different concentrations of TWS119. The data were expressed as the mean ± SD. Figure S6. (A–C) Quantitative analysis of the relative grey levels of pSer9-GSK-3β and GSK-3β after TWS119 treatment. (D–E) Quantitative analysis of the relative grey levels of Gli1 after TWS119 treatment. (F–H) Quantitative analysis of the relative grey levels of Runx2, Osterix and OCN. All data were expressed as the mean ± SD. *p < 0.05, **p < 0.01, ns: no significance. Figure S7. (A) Cell viability after incubation for 3 days with different concentrations of GANT58 was assessed using a CCK-8 kit, *p < 0.05, compared with the 0 μM group. (B) Cell inhibition rate after incubation for 3 days with different concentrations of GANT58. Figure S8. (A) Representative images of H&E staining of lung, liver and kidney collected from the four groups. Table S1. [file 12951_2022_1351_MOESM1_ESM.docx]

**Additional file**

**GSK-3β suppression upregulates Gli1 to alleviate osteogenesis inhibition in titanium nanoparticle-induced osteolysis.**

**The name(s) of the author(s)**

*Qing Wang^a,1^; Wei Zhang^a,1^; Xiaole Peng^a,1^; Yunxia Tao^a^; Ye Gu^c^; Wenming Li^a^; Xiaolong Liang^a^; Liangliang Wang^a,d^; Zerui Wu^a,e^; Tianhao Wang^a^; Haifeng Zhang^a^; Xin Liu^a^; Yaozeng Xu^a,^ *; Yu Liu^b,^ *; Jun Zhou^a,^ *; Dechun Geng^a,^ **

**Name and address of the institution**

a: Department of Orthopaedics, The First Affiliated Hospital of Soochow University, Suzhou, 215006, China.

b: Department of Orthopaedics, Wuxi Ninth People's Hospital Affiliated to Soochow University, Wuxi, 214062, China.

c: Department of Orthopaedics, Changshu Hospital Affiliated to Soochow University, First People’s Hospital of Changshu City, Changshu, China

d: Department of Orthopaedics, The Affiliated Changzhou No. 2 People’s Hospital of Nanjing Medical University, Changzhou, PR China

e: Department of Orthopaedics, The Affiliated Hospital of Xuzhou Medical University, Xuzhou, China

^1^ **Contribute equally to this work**

*** Correspondence and requests for materials should be addressed to**

szgengdc@suda.edu.cn (D. Geng); wxsjyly@126.com (Y. Liu);

xuyaozeng@163.com (Y. Xu); zhou.jun.roy@hotmail.com (J. Zhou).

**Disclosures**

The authors have declared that no competing interest exists.


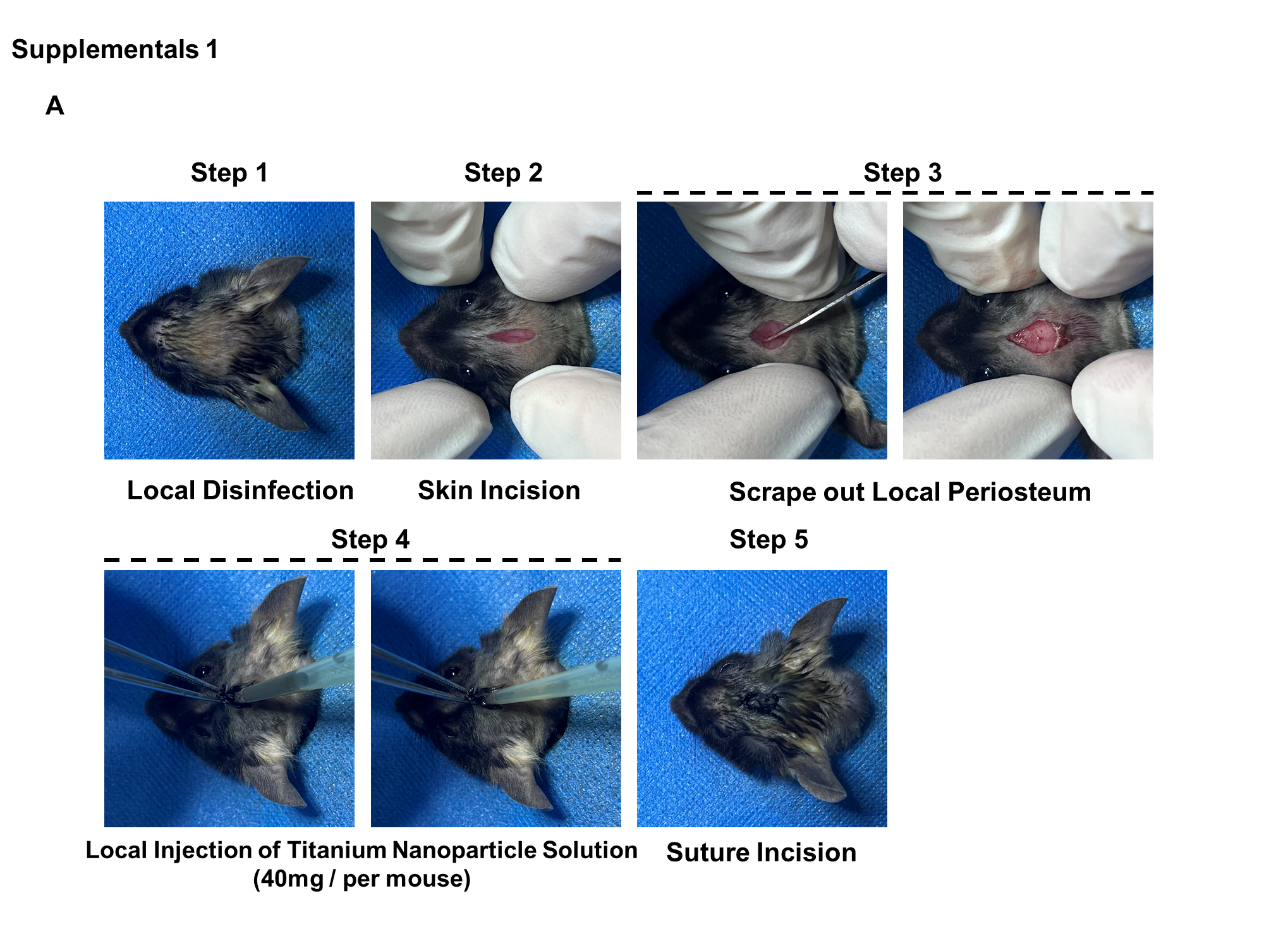


**Fig. S1.** (A) The detailed surgical steps of the particle-implanted murine calvaria model.


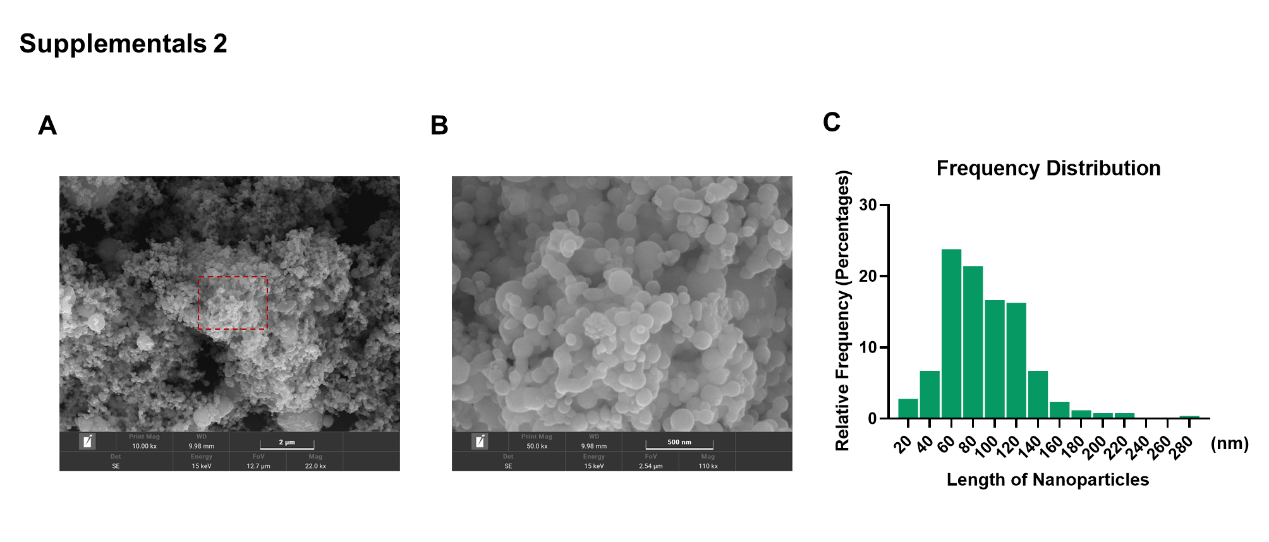


**Fig. S2.** (A-B) Representative Scanning electron microscopy (SEM) image of Ti nanoparticles. (C) Frequency distribution of Ti nanoparticles size.


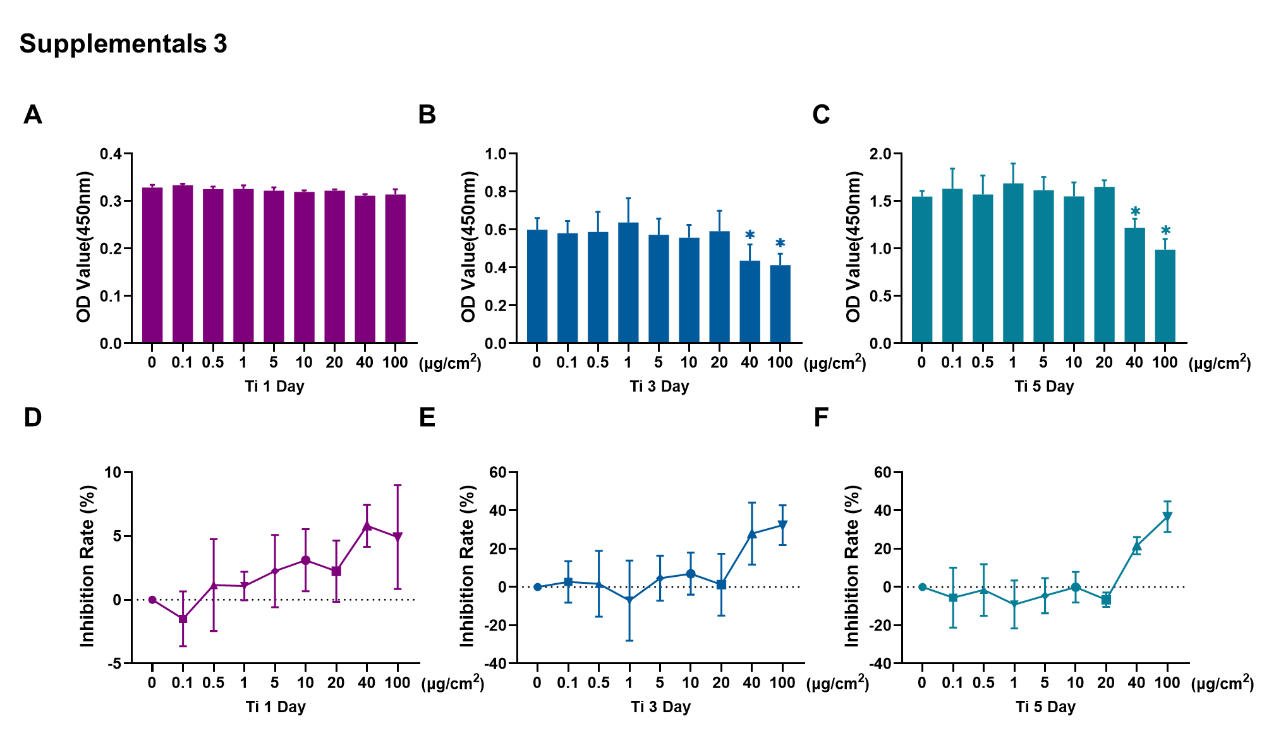


**Fig. S3.** (A-C) Cell viability after incubation for 1, 3 and 5 days with different concentrations of Ti particles was assessed using a CCK-8 kit, *p < 0.05, compared with the 0 μg/cm^2^ group. (D-F) Cell inhibition rate after incubation for 1, 3 and 5 days with different concentrations of Ti particles. The data were expressed as the mean ± SD.


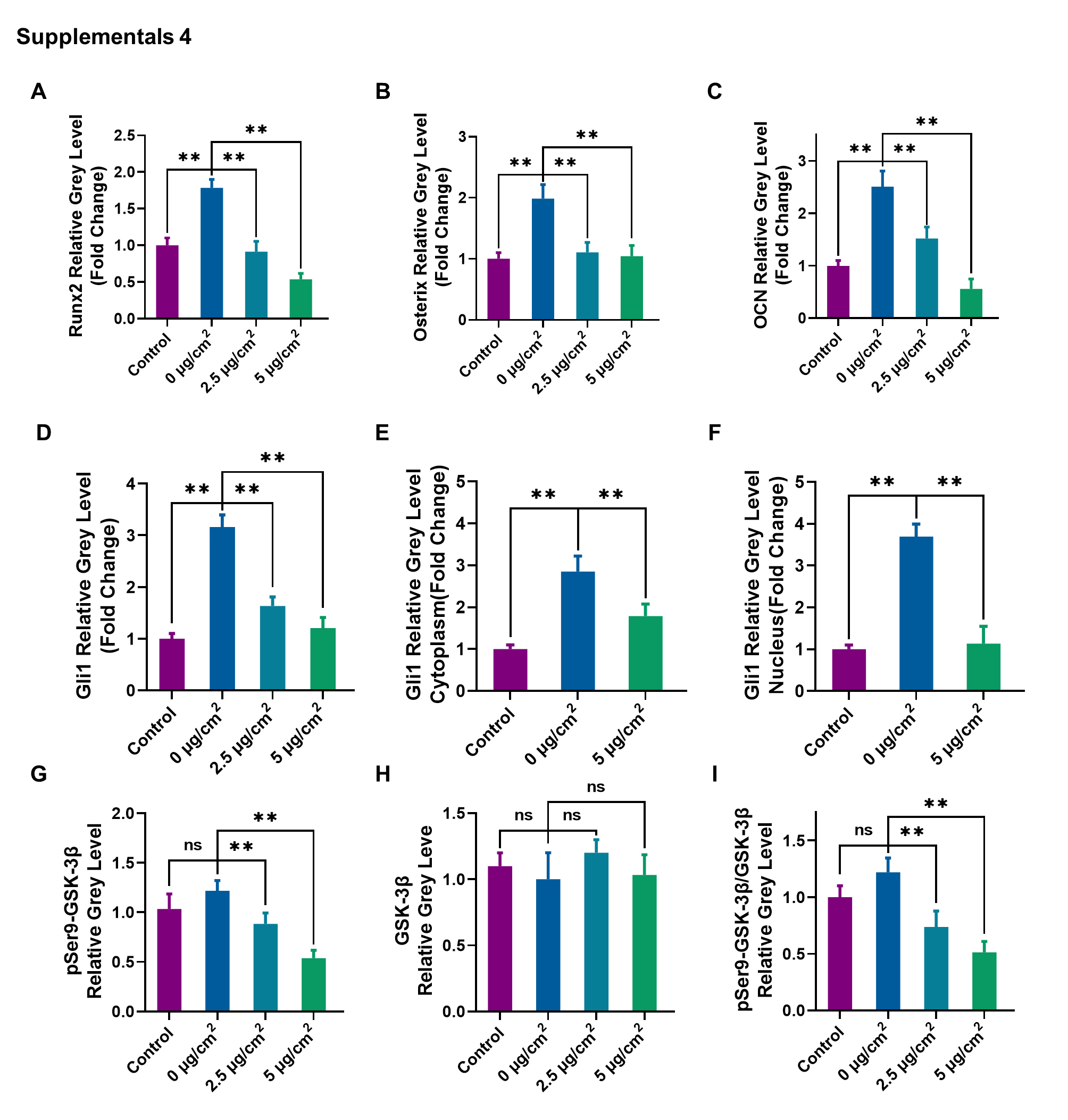


**Fig. S4.** (A-C) Quantitative analysis of the relative grey levels of Runx2, Osterix and OCN. (D-F) Quantitative analysis of the relative grey levels of Gli1. (G-I) Quantitative analysis of the relative grey levels of pSer9-GSK-3β and total GSK-3β. All data were expressed as the mean ± SD. *p < 0.05, **p < 0.01, ns: no significance.


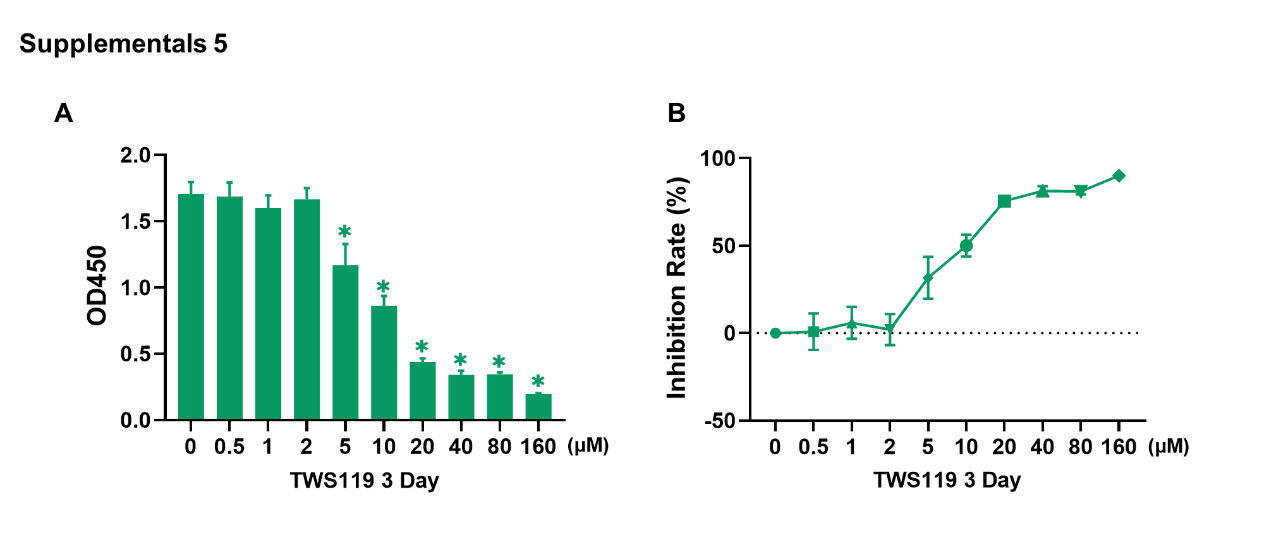


**Fig. S5.** (A) Cell viability after incubation for 3 days with different concentrations of TWS119 was assessed using a CCK-8 kit, *p < 0.05, compared with the 0 μM group. (B) Cell inhibition rate after incubation for 3 days with different concentrations of TWS119. The data were expressed as the mean ± SD.


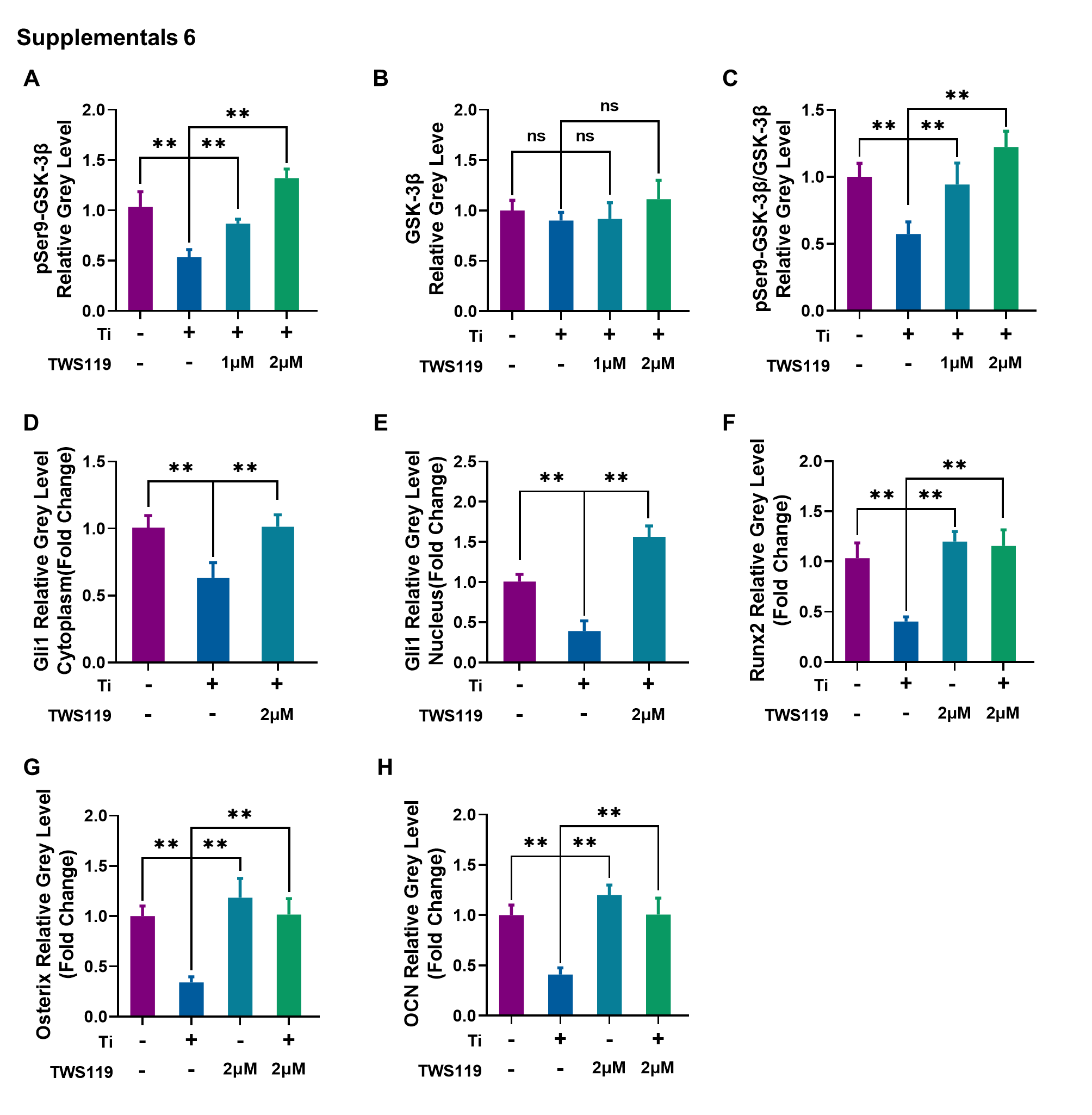


**Fig. S6.** (A-C) Quantitative analysis of the relative grey levels of pSer9-GSK-3β and total GSK-3β after TWS119 treatment. (D-E) Quantitative analysis of the relative grey levels of Gli1 after TWS119 treatment. (F-H) Quantitative analysis of the relative grey levels of Runx2, Osterix and OCN. All data were expressed as the mean ± SD. *p < 0.05, **p < 0.01, ns: no significance.


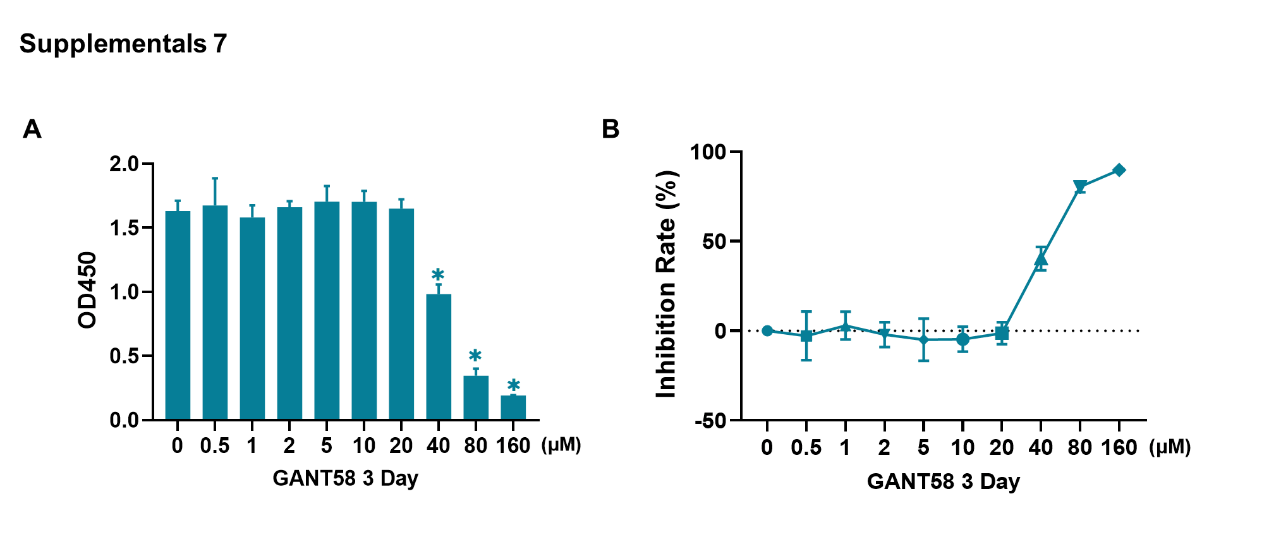


**Fig. S7.** (A) Cell viability after incubation for 3 days with different concentrations of GANT58 was assessed using a CCK-8 kit, *p < 0.05, compared with the 0 μM group. (B) Cell inhibition rate after incubation for 3 days with different concentrations of GANT58.


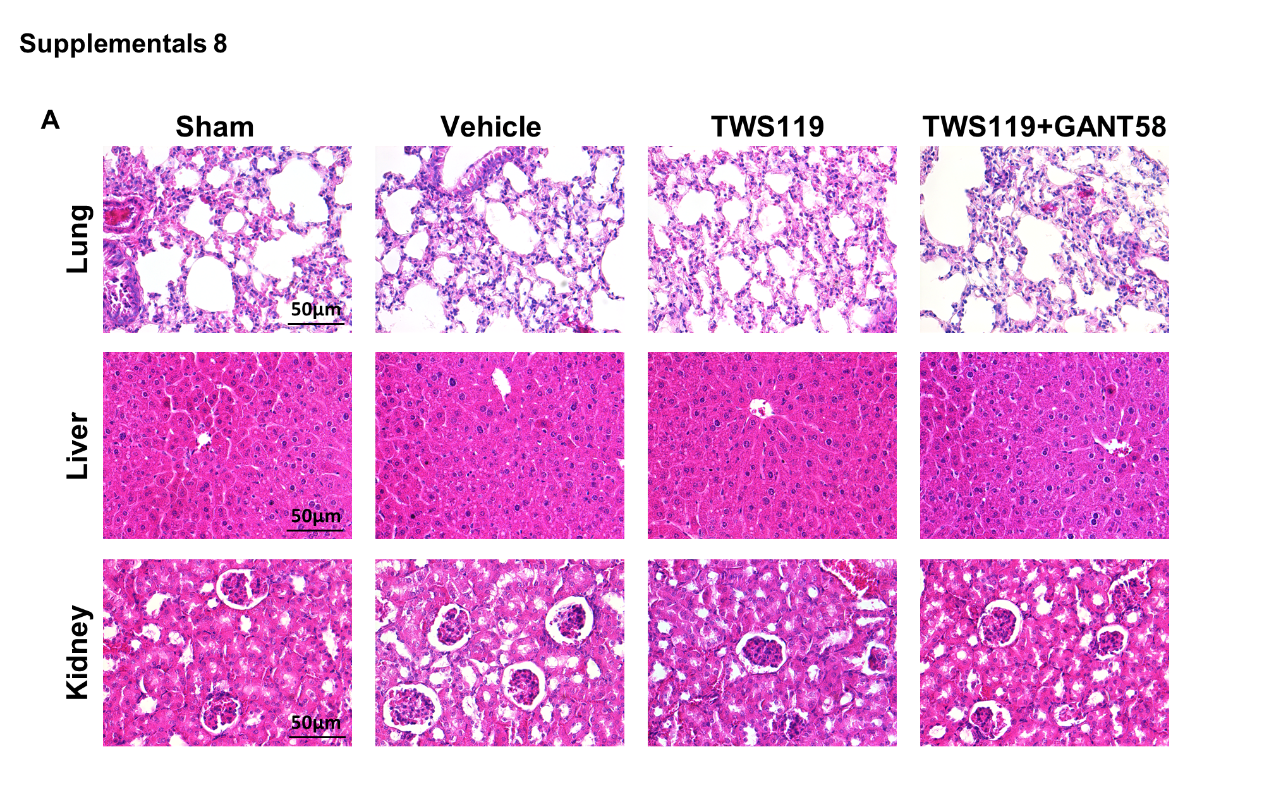


**Fig. S8.** (A) Representative images of H&E staining of lung, liver and kidney collected from the four groups.

**Table S1. Primer sequence**

| Gene (mus) | Forward Primers (5’-3’) | Reverse Primers (5’-3’) |
| --- | --- | --- |
| GAPDH | GGTTGTCTCCTGCGACTTCA | TGGTCCAGGGTTTCTTACTCC |
| Runx2 | CCTTCAAGGTTGTAGCCCTC | GGAGTAGTTCTCATCATTCCCG |
| OCN | TTGAACTGTTTGTTTTGGACCC | CCAACAGACACCAGTTGTAAAG |
| Osterix | TCGTCTGACTGCCTGCCTAGTG | CTGCGTGGATGCCTGCCTTG |
| Gli1 | GGTGTGTAATTACGTTCAGTCG | GGATAGGAGCCTGATTTGTGAT |

**Abbreviations:** GAPDH: glyceraldehyde 3-phosphate dehydrogenase; Runx2: Runt-related transcription factor 2; OCN: Osteocalcin.
